# Differential expression and functional analysis of circular RNA in ovaries of Tibetan sheep with different fecundity

**DOI:** 10.1515/biol-2025-1256

**Published:** 2026-05-04

**Authors:** Yunxin Jin, Wu Sun, Xiayang Jin, Shike Ma

**Affiliations:** Academy of Animal Science and Veterinary Medicine, Qinghai University, Xining, 810016, China; Key Laboratory of Livestock and Poultry Genetics and Breeding on the Qinghai-Tibet Plateau, Ministry of Agriculture and Rural Affairs, Xining, 810016, China; Plateau Livestock Genetic Resources Protection and Innovative Utilization Key Laboratory of Qinghai Province, Xining, 810016, China

**Keywords:** Tibetan sheep, ovary, circRNA, fertility, GO, KEGG

## Abstract

This study represents one of the first investigations of circular RNAs (circRNAs) in the ovaries of Tibetan sheep, with a focus on their potential role in reproductive performance. The aim was to identify circRNAs involved in ovarian development and fertility regulation. High-throughput sequencing was applied to analyze ovarian tissue samples from 20 Tibetan sheep, leading to the identification of 82 differentially expressed circRNAs between the single multiparous (SL) and multiple multiparous (ML) groups, with 35 upregulated and 47 downregulated circRNAs. Gene ontology (GO) and Kyoto Encyclopedia of Genes and Genomes (KEGG) pathway enrichment analyses revealed significant associations with pathways involved in cell growth, cell cycle progression, energy metabolism, hormone regulation, and cellular transport. Protein-protein interaction (PPI) analysis identified key target genes, including *EP300*, *CUL1*, and *oar-miR-654-5p*. A comprehensive quaternary regulatory network, integrating circRNA, miRNA, mRNA, and related pathways, was constructed. This study provides novel insights into the molecular mechanisms underlying fertility in Tibetan sheep and identifies potential molecular targets for improving reproductive biotechnology. These findings lay the foundation for future molecular breeding programs aimed at enhancing fertility in Tibetan sheep.

## 1 Introduction

Tibetan sheep (*Ovis aries*) represent a critically important indigenous livestock genetic resource in China, predominantly inhabiting the Qinghai-Tibetan Plateau region. The meat of Tibetan sheep exhibits excellent palatability characterized by fine muscle fiber texture [1], and demonstrates unique nutritional properties including abundant essential amino acids, vitamins, and trace mineral elements (iron, zinc, and selenium) that distinguish it from other mutton varieties [2], serving as a vital source of high-quality animal protein for local communities. As an economically crucial livestock species in alpine ecosystems, the reproductive performance of Tibetan sheep directly influences the economic sustainability of local pastoral industries.

The lambing process in sheep is a highly complex and tightly regulated biological event, influenced by a wide range of genetic and environmental factors. Reproductive performance in sheep is governed by polygenic regulatory mechanisms, where multiple genes contribute to various stages of reproductive physiology, including follicular development, ovulation, and embryo implantation. One of the key genes involved in regulating fertility is the *FecB* gene (*BMPR1B*), which has been shown to enhance both the ovulation rate and litter size through its influence on the development of ovarian follicles [3], 4]. Additionally, the *GDF9* and *BMP15* genes work synergistically to regulate oocyte maturation and ovulatory competence by modulating granulosa cell functions, which are essential for successful ovulation [5], [6], [7]. Furthermore, polymorphisms in the *ESR* (Estrogen Receptor) gene significantly affect the responsiveness to reproductive hormones and uterine receptivity, influencing the embryo’s ability to implant and develop in the uterus [8], [9], [10], [11].

Although these genes are crucial for regulating fertility, there is significant variation in reproductive performance between monotocous (single-lamb) and polytocous (multiple-lamb) populations of Tibetan sheep. This variation arises from a multifactorial regulatory system that involves not only the regulation of ovarian follicular dynamics [11], but also the coordination of the hypothalamic-pituitary-gonadal (HPG) axis, which governs hormonal signaling for reproduction [12]. Additionally, epigenetic factors, such as DNA methylation, play a role in regulating the expression of fertility-related genes, which may contribute to the observed fertility differences [13].

Furthermore, Tibetan sheep, living at high altitudes, face unique environmental challenges such as hypoxic stress, which can negatively affect reproductive function. Low oxygen and oxidative stress in high-altitude environments have been shown to lead to intrauterine growth retardation. Studies have shown that the administration of antioxidant vitamins C and E significantly increases plasma progesterone and 17*β*-oestradiol levels in pregnant ewes, supporting normal fetal development, particularly in sheep not adapted to high-altitude conditions. While fetal sheep can partially mitigate the effects of hypoxia through adaptive responses such as increased hemoglobin concentration, these compensatory measures are insufficient to fully prevent growth retardation [14], 15]. These high-altitude conditions have driven adaptations in Tibetan sheep at the genetic and molecular levels, potentially influencing fertility-related pathways regulated by circRNAs and other non-coding RNAs, thus highlighting the complex interplay between genetics, environment, and reproductive performance.

Circular RNAs (circRNAs) are a class of covalently closed, single-stranded non-coding RNA molecules formed through back-splicing of precursor mRNA in eukaryotic organisms [16]. These molecules, which constitute prevalent nonlinear RNA architectures, are widespread across species and have gained attention due to their versatile regulatory functions in a variety of biological processes. Recent advances in RNA sequencing and bioinformatics have led to the systematic identification of numerous circRNAs involved in germ cell development across various mammalian species [17]. CircRNAs exert multifaceted biological functions primarily through mechanisms such as acting as miRNA sponges to regulate target gene expression [18], [19], [20], interacting with RNA-binding proteins (RBPs) to influence post-transcriptional regulation [21], 22], stabilizing mRNA transcripts via structural interactions [23], and serving as templates for functional peptide synthesis under certain conditions [24], 25].

In mammalian models, such as bovine, porcine, and murine species, circRNAs have been shown to play crucial roles in ovarian function. For example, porcine circ*0001651* and circ*0015292* exhibit expression patterns associated with follicular development through transcriptional regulation of granulosa cell genes [26], 27]. The circ*FSHR* molecule enhances follicle-stimulating hormone (FSH) signaling by feedback regulation of FSHR expression [28], while circ*BMP15* participates in oocyte maturation through modulation of the BMP/Smad signaling pathway [29]. Although research on circRNAs in sheep is still limited, studies have revealed their potential role in fertility regulation. One study identified 38,979 circRNAs in Xiaowei Han sheep follicles, showing differential expression between *FecB* homozygous (BB) and wild-type (WW) genotypes, with involvement in hormone-related pathways such as MAPK, gap junctions, and progesterone-mediated oocyte maturation [28]. Additionally, 129 miRNAs were predicted to interact with 336 differentially expressed circRNAs, with follicle-specific circRNAs like oar_circ_*0000523* and oar_circ_*0028984* potentially regulating LH synthesis. Another study identified 147 and 364 differentially expressed circRNAs in sheep uteri during the follicular and luteal phases, respectively, involved in estrogen, TGFβ, GnRH signaling pathways [29]. These findings highlight the emerging role of circRNAs in sheep fertility, but research, particularly in Tibetan sheep, remains limited.

We hypothesize that the fertility differences between single-lamb and multiple-lamb Tibetan sheep may be linked to differential circRNA expression patterns. Using high-throughput sequencing and functional annotation, we identified differentially expressed circRNAs in the ovaries of Tibetan sheep with high and lowfecundity, revealing key circRNA-mediated pathways that regulate fertility, particularly between single-lamb and multiple-lamb individuals. We also constructed a regulatory network involving circRNAs, miRNAs, mRNAs, and pathways, providing a novel insight into the molecular mechanisms underlying fertility in Tibetan sheep. Our findings offer new molecular targets for improving breeding strategies aimed at enhancing the reproductive efficiency of plateau-adapted sheep populations.

## 2 Materials and methods

### 2.1 Experimental animals and grouping

A total of 20 clinically healthy, multiparous (3–4 years old) Tibetan ewes with an average body weight of 54.45 ± 1.25 kg were selected from the High-Altitude Ecological Animal Husbandry Science and Technology Demonstration Park, Haibei Tibetan Autonomous Prefecture, Qinghai Province, China (37°06′N, 100°91′E; altitude 3,200–3,500 m). All ewes were raised under a uniform grazing plus supplementary feeding management system and exhibited normal estrous cycles. According to continuous lambing records over the previous three years, the ewes were divided into two distinct phenotypic groups: a high-fecundity multiple-lamb group (ML, *n* = 10, average litter size ≥ 2.0) and a low-fecundity single-lamb group (SL, *n* = 10, litter size = 1.0 in all recorded parities). Individual ewes were identified as ML1–ML10 and SL1–SL10. To eliminate variation caused by different estrous cycle stages, estrus synchronization was performed in November 2024 using intravaginal progesterone-releasing devices (CIDR, 1.38 g progesterone; Zoetis, USA) for 12 days.

Upon CIDR removal (Day 12), each ewe received an intramuscular injection of 0.1 mg cloprostenol sodium (PGF2*α*, Ningbo Sansheng Pharmaceutical Co., Ltd., China) and, 6–8 h later, 350 IU pregnant mare serum gonadotropin (PMSG, Ningbo Sansheng Pharmaceutical Co., Ltd., China). Ovaries were collected on days 2–3 of the third synchronized estrous cycle (36–72 h after CIDR withdrawal), when preovulatory follicular development is most active and fecundity-related differences are maximized.

Ewes were slaughtered by exsanguination following electrical stunning in accordance with local abattoir protocols. Both left and right ovaries were excised within 15 min post-slaughter, immediately rinsed in ice-cold sterile saline, gently blotted dry, and snap-frozen in liquid nitrogen in the field. The two ovaries from the same individual were pooled as one biological replicate. Samples were transported in liquid nitrogen to the laboratory and stored at −80 °C until total RNA extraction. All animal procedures were conducted in strict accordance with the Guidelines for the Care and Use of Laboratory Animals of the Ministry of Science and Technology of China.

**Ethical approval:** The research related to animal use has been complied with all the relevant national regulations and institutional policies for the care and use of animals, and has been approved by the Animal Ethics and Use Committee of Qinghai University.

### 2.2 RNA extraction and library preparation

Total RNA was extracted from pooled bilateral ovarian tissues of Tibetan sheep using TRIzol Reagent (Invitrogen, USA) with on-column DNase I digestion (QIAGEN, Germany). RNA concentration and purity were assessed using a NanoDrop ND-2000 spectrophotometer, and integrity was evaluated with an Agilent 2,100 Bioanalyzer RNA 6000 Nano Kit (Agilent Technologies, USA). Only samples exhibiting an RNA Integrity Number (RIN) ≥ 8.5, 28S/18S ratio ≥ 1.1, and A260/A280 between 1.9 and 2.1 were selected for library construction. All samples exhibited high RNA integrity with an average RIN of 9.15 ± 0.28 (range 8.7–9.6) and 28S/18S ratio of 1.39 ± 0.18 (Supplementary Table 1).

Ribosomal RNA was depleted using the Ribo-Zero™ rRNA Removal Kit (Illumina, USA). Linear RNAs were digested with RNase R (20 U/μg total RNA; Epicentre, USA) at 37 °C for 30 min to enrich circRNAs, followed by purification with the RNeasy MinElute Cleanup Kit (QIAGEN, Germany). Libraries were prepared using the NEBNext^®^ Ultra™ II Directional RNA Library Prep Kit (New England Biolabs, USA). Library quality was assessed using an Agilent 2,100 Bioanalyzer High Sensitivity DNA Kit and Qubit 4.0 Fluorometer. Libraries were deemed qualified only if they met all of the following criteria: (i) a single dominant peak at 300–500 bp, (ii) absence of adaptor/primer dimers (<250 bp), (iii) no low-molecular-weight fragments or bubbles, (iv) concentration ≥ 10 ng/μL, and (v) molarity ≥ 2 nM. All 20 libraries satisfied these criteria (representative sequencing quality control profiles are shown in Supplementary Figure 1). Qualified libraries were sequenced on an Illumina NovaSeq 6,000 platform in 150 bp paired-end mode at Annoroad Gene Technology (Beijing, China).

### 2.3 Sequencing data evaluation

#### 2.3.1 Sequencing data filtering

The raw sequencing data underwent rigorous quality control processing through Fastp (version 0.23.2; BGI, Shenzhen, China) to eliminate low-quality reads (containing >20 % bases with Phred scores <10), adapter-contaminated reads, and reads with >5 % ambiguous nucleotides (*N*), ultimately yielding high-quality clean reads.

#### 2.3.2 Removing ribosomal RNA

Residual rRNA reads were removed using SOAP2 [30] to minimize contamination effects on downstream circRNA analysis.

#### 2.3.3 Reference genome alignment

Clean reads were mapped to the Tibetan sheep (*O. aries*) reference genome (assembly: Oar_ v4.0) using BWA-MEM (version 0.7.12-r1039) [31] with the parameters: bwa mem *-Chigh_strigency – Cmapq_uni 10*.

### 2.4 CircRNA prediction

CircRNAs were predicted from the clean reads using CIRI software [32] (version 2.0.2) based on the detection of back-spliced junction (BSJ) reads, which identify circularization sites. The analysis was conducted with the following parameters: *Cmax_span = 200,000, Cthread_num = 2, and CchrM = chrM* to exclude mitochondrial genome interference.

#### 2.4.1 Alternative splicing analysis

Exon positional annotation and alternative splicing analysis of predicted circRNAs were performed using CIRI-AS software [33] (version 1.2) with default parameters. This enabled precise structural characterization of circRNAs, exploration of their diversity and splicing variations, thereby providing foundational data for subsequent functional investigations.

#### 2.4.2 Identification of source genes

The source genes of circRNAs were identified based on their genomic positional information, allowing for precise localization of their parental genes. This facilitated subsequent functional annotation to elucidate their potential biological roles.

### 2.5 CircRNA expression quantification

CircRNA expression levels were quantified based on back-spliced junction (BSJ) reads following the Spliced Reads Per Billion Mapped (SRPBM) normalization method [34]. Specifically, BSJ read counts were adjusted against the total mapped reads per sample and normalized by sequencing depth and average read length to ensure cross-sample comparability. to account for sequencing depth and read length variability.

### 2.6 Correlation analysis of CircRNA samples

Principal component analysis (PCA) and Pearson correlation analysis were conducted using the expression data of each sample to evaluate sample consistency. The correlation between samples was visualized with heatmaps, and hierarchical clustering was used to investigate sample relationships.

### 2.7 Cluster analysis

To analyse differential circRNA expression between experimental groups and to visualise circRNA expression patterns, heatmaps were generated based on normalised FPKM (Fragments Per Kilobase of transcript per Million mapped reads) values. Z-score transformation was applied to the expression data prior to plotting, with colour gradients indicating relative expression levels – red representing high expression and blue representing low expression. Data processing and visualisation were performed using the *pheatmap* or *ComplexHeatmap* packages in R.

### 2.8 CircRNA differential expression analysis

Differential expression analysis of circRNAs and their host genes was performed using DESeq2 (version 1.38.0 in R version 4.3.2 [35]. Raw read counts were normalized using the variance-stabilizing transformation (VST) implemented in DESeq2. Multiple testing correction was conducted using the Benjamini–Hochberg false discovery rate (FDR) procedure. Differentially expressed circRNAs were identified based on an absolute log_2_ fold change ≥ 1 and adjusted p-value (FDR) < 0.05.

To evaluate and correct for potential batch effects, surrogate variable analysis was performed using the sva package (v3.46.0). The ComBat-seq function from the sva package was applied to the raw count matrix to remove both known (e.g., sequencing lane and library preparation date) and unknown batch effects. Principal component analysis (PCA) on VST-normalized counts was subsequently conducted using the plotPCA function in DESeq2 to visualize sample relationships.

### 2.9 Functional enrichment and protein interaction network analysis of differential CircRNA host genes

In order to fully reveal the biological function of differentially expressed circRNA host genes and their potential role in regulating ovarian development, GO functional annotation analysis, KEGG pathway enrichment analysis and protein interaction network (PPI) analysis were carried out in turn according to the standard bioinformatics process. Gene Ontology (GO) enrichment analysis was performed using the Gene Ontology database (release 2025-05-01, accessed on May 28, 2025; http://geneontology.org/).KEGG pathway enrichment analysis was conducted using the KEGG database (Release 114.0, accessed on June 2, 2025; https://www.kegg.jp/).Protein–protein interaction (PPI) networks were constructed using the STRING database (version 11.5, accessed on June 10, 2025; https://string-db.org/).

#### 2.9.1 GO and KEGG enrichment analysis

Differentially expressed genes (DEGs) were mapped to Gene Ontology (GO) terms in the GO database through enrichment analysis. The number of DEGs associated with each GO term was calculated to identify significantly enriched functional categories. A list of DEGs associated with specific GO functions was obtained to explore the biological processes, cellular components, and molecular functions most relevant to the observed expression changes.

KEGG pathway enrichment analysis was performed to identify pathways significantly overrepresented among the differentially expressed genes (DEGs) compared with the background gene set, using the hypergeometric test. This analysis was used to evaluate the major biochemical metabolic pathways and signal transduction pathways associated with the DEGs. KEGG enrichment analysis was performed using the *clusterProfiler* package in R. The *q*-value represents the p-value adjusted for multiple hypothesis testing, with a range between 0 and 1. A smaller *q*-value indicates a higher level of statistical significance for pathway enrichment.

#### 2.9.2 Protein-protein interaction network analysis

Based on functional enrichment analysis, to further identify potential key regulatory nodes (hub genes), the list of host genes of differentially expressed circRNAs was imported into the STRING database to construct a protein–protein interaction (PPI) network. The parameters were set as follows: species was set to *O. aries* (sheep); the minimum required interaction score was 0.15 (low confidence); the network was constructed based on evidence from experiments, curated databases, co-expression, and gene neighborhood information; and disconnected nodes were hidden. The resulting PPI network was exported in TSV format and imported into Cytoscape software (v3.10.3) for visualization and further analysis. The CytoNCA plugin (v2.1.6) was used to identify hub genes based on a combined evaluation of Betweenness Centrality (BC), Degree Centrality (DC), and Eigenvector Centrality (EC), which served as prioritized candidates for subsequent functional validation and regulatory analysis.

### 2.10 Targeted prediction analysis and construction of CircRNA-miRNA-mRNA-pathway quaternary regulatory network

After obtaining the differential expression results of circRNAs, target miRNAs potentially bound by circRNAs were predicted using three software tools: In-taRNA [36], miRanda [37], and PITA3 [38]. Venn diagrams were generated to visualize the overlap of the results from these different algorithms, and the intersecting results from the final two software tools were selected as candidate miRNA-circRNA pairs. Based on the RNA-ceRNA regulatory network, miRNA target mRNAs were then predicted using TargetScan [39], miRanda, and PITA, and the intersection of the results from these three software tools was taken to define candidate miRNA-mRNA pairs, as visualized by a Venn diagram.

#### 2.10.1 GO and pathway functional enrichment analysis of miRNA target genes

The target genes were mapped to the Gene Ontology (GO) database to calculate the number of genes associated with each GO term. Enrichment analysis was conducted using the *phyper* function in R software, identifying significantly enriched terms in the target genes compared to the entire genome. To further investigate the biological functions of miRNA-targeted genes, GO analysis was performed, categorizing the genes into three major functional categories: Biological Process (BP), Cellular Component (CC), and Molecular Function (MF). Pathway enrichment analysis was performed using KEGG pathways as the reference, applying a hypergeometric test to identify pathways in which the target genes were significantly enriched compared to all annotated genes. The target genes were then classified into relevant biological pathways. Enrichment analysis was conducted using the *phyper* function in R software, and the resulting *p*-values were corrected for false discovery rate (FDR), with an FDR threshold of ≤ 0.01 considered as significantly enriched. Finally, the most important biochemical metabolic and signal transduction pathways associated with the target genes were identified.

#### 2.10.2 Prediction of targeted gene-related regulatory networks

To further identify the potential key regulatory nodes and screen the predicted core CircRNA-miRNA and miRNA-mRNA prediction relationships, the screened candidate miRNA-circRNA pairing relationship, candidate miRNA-mRNA pairing relationship and mRNA Pathway enrichment results (the most significant enrichment of the first three reproductive-related pathways) were summarized into the same table, and Cytoscape software was introduced in csv format to draw the prediction-related core regulatory network.

## 3 Results and analyses

### 3.1 Results of the CircRNA sequencing data quality assessment

In this study, we performed circRNA sequencing on ovarian tissue from Tibetan sheep (*O. aries*) using high-throughput technology to generate high-quality transcriptomic data. Raw sequencing reads were assessed for quality before bioinformatic analysis to measure error rates and ensure data reliability. Key parameters, such as total read counts, base quality (Q20/Q30), and GC content, were evaluated across all samples. Quality control, applied through the fastp pipeline, removed adapter contamination, low-quality reads (Phred score < 20), and reads with high ambiguity (N > 5 %). After filtering, each sample had 78–103 million clean reads, corresponding to 11–15 gigabases of high-confidence data. The base-call accuracy was high, with Q20 ≥ 96.72 % and Q30 ≥ 90.88 %, and GC content was within the expected range of 45.17–50.89 % (Table 1). These metrics confirmed the sequencing data’s technical robustness. Moreover, reference genome alignment efficiency exceeded 99.8 % for all samples against the *O. aries* genome (*Oar_v4.0*; Table 2), ensuring reliable mapping for subsequent analyses, including circRNA identification, differential expression, and functional annotation.

**Table 1:** Quality statistics of reads after filtering.

Sample	Clean reads(*M*)	Clean bases(*G*)	Q20(%)	Q30(%)	GC(%)	Read Length(bp)
ML1	82.4536M	12.3680G	97.40	92.58	49.07	150
ML10	91.1177M	13.6676G	97.95	93.55	45.17	150
ML2	97.6820M	14.6523G	97.03	91.71	48.79	150
ML3	101.8451M	15.2768G	97.36	92.53	48.49	150
ML4	100.4161M	15.0624G	97.41	92.49	48.24	150
ML5	86.5301M	12.9795G	96.92	91.40	47.98	150
ML6	99.5202M	14.9280G	97.08	91.72	49.14	150
ML7	103.3107M	15.4966G	97.47	92.60	48.60	150
ML8	98.9434M	14.8415G	97.46	92.44	48.96	150
ML9	78.4072M	11.7611G	97.19	91.94	47.48	150
SL1	86.9242M	13.0386G	96.77	91.35	50.89	150
SL10	99.9592M	14.9939G	97.28	92.21	47.97	150
SL2	79.7140M	11.9571G	97.34	92.40	48.92	150
SL3	103.2831M	15.4925G	97.49	92.75	46.93	150
SL4	98.8611M	14.8292G	97.31	92.33	48.92	150
SL5	79.2243M	11.8836G	96.72	90.88	48.39	150
SL6	102.5764M	15.3865G	97.19	92.11	48.92	150
SL7	84.8939M	12.7341G	96.90	91.43	48.31	150
SL8	89.3595M	13.4039G	96.87	91.31	47.70	150
SL9	97.4390M	14.6159G	97.14	91.96	47.42	150

**Table 2:** Statistics of reference genome alignment.

Sample	Total reads	Mapped reads	Mapped rate (%)
ML1	82453326	82390479	99.92 %
ML10	91117608	91093983	99.97 %
ML2	97681336	97545873	99.86 %
ML3	101844948	101718755	99.88 %
ML4	100416018	100348790	99.93 %
ML5	86529368	86411525	99.86 %
ML6	99519880	99374202	99.85 %
ML7	103310402	103272440	99.96 %
ML8	98943006	98880995	99.94 %
ML9	78407116	78248701	99.80 %
SL1	86924028	86224887	99.20 %
SL10	99958916	99804840	99.85 %
SL2	79713906	79531675	99.77 %
SL3	103282896	103195550	99.92 %
SL4	98860940	98821373	99.96 %
SL5	79224146	79187555	99.95 %
SL6	102576206	102531211	99.96 %
SL7	84893806	84720480	99.80 %
SL8	89359450	89151252	99.77 %
SL9	97438884	97304438	99.86 %

The high sequencing quality (average Q30 > 92 %) and high mapping rate (>92 % to the *O. aries* reference genome, ARS-UI_Ramb_v2.0“*Oar_v4.0*”) ensured reliable downstream circRNA identification and quantification. A greater number of circRNAs and higher splicing diversity were detected in ovaries of the single-lamb (SL) group compared to the multiple-lamb (ML) group. This unexpected higher circRNA complexity in the lower-fecundity group suggests that circRNAs may act as fine-tuned negative regulators of fecundity in Tibetan sheep, potentially through miRNA sponging or interaction with RNA-binding proteins that modulate follicular development and ovulation rate. These findings highlight a complex regulatory role of circRNAs in ovarian function and provide a novel perspective on the molecular mechanisms underlying variation in litter size in high-altitude-adapted sheep.

### 3.2 Correlation and PCA analysis of samples

**Figure 1:**
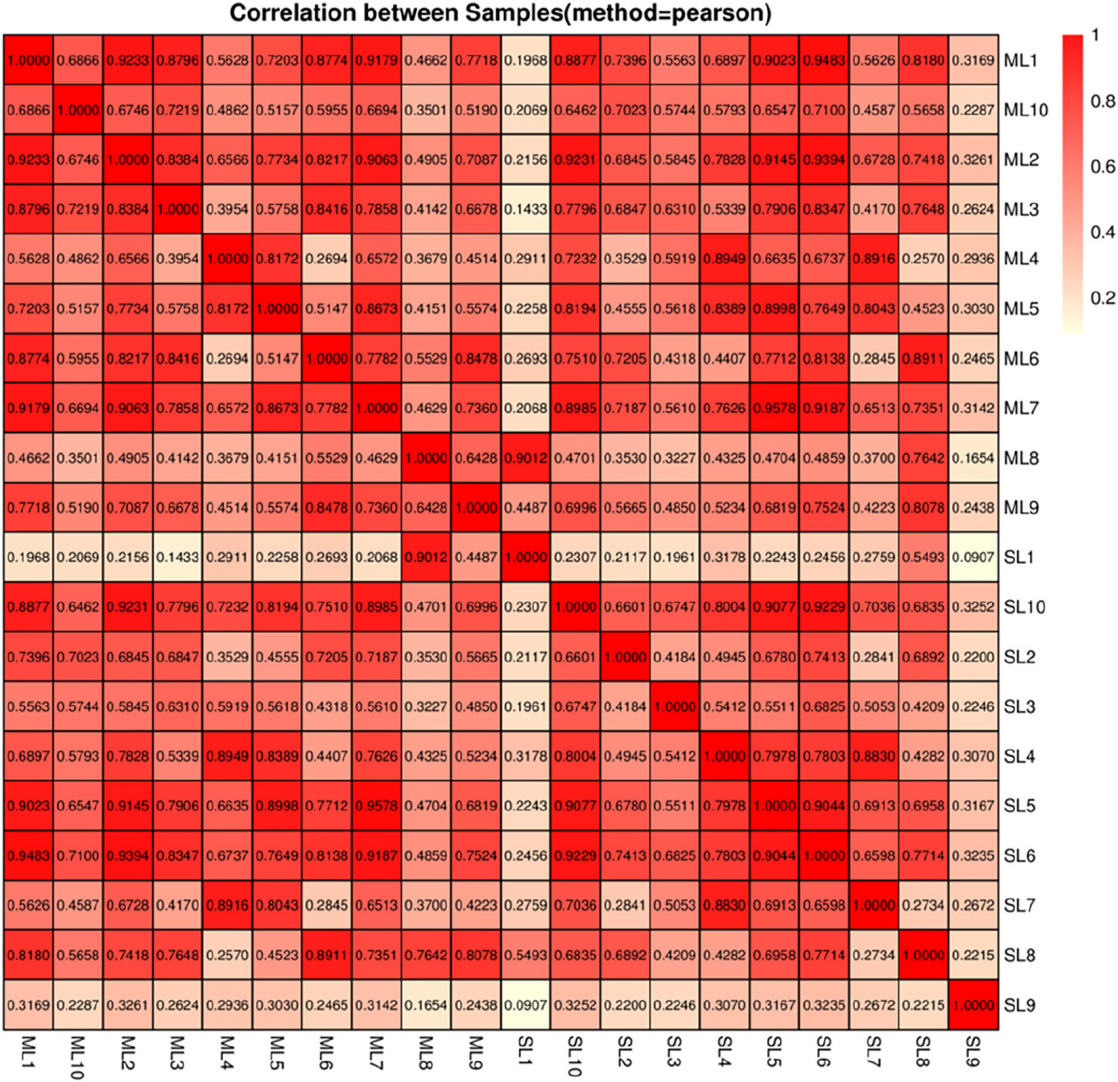
Heatmap of sample-to-sample correlations for 20 samples.

To assess data reproducibility and sample relationships, Pearson correlation coefficients were calculated based on variance-stabilized circRNA expression profiles (Figure 1). The analysis confirmed a general trend of intra-group transcriptional consistency, with mean correlation coefficients of *r = 0.724* ± *0.112* for the ML group and *r = 0.689* ± *0.135* for the SL group^1^. Although specific individuals exhibited relative divergence (e.g., SL1 and ML8) or higher cross-group correlation (e.g., SL10), likely reflecting the inherent biological heterogeneity within ovarian tissues, approximately 72.4 % of within-group replicate pairs maintained strong positive correlations (*r > 0.7*)^2^.

**Figure 2:**
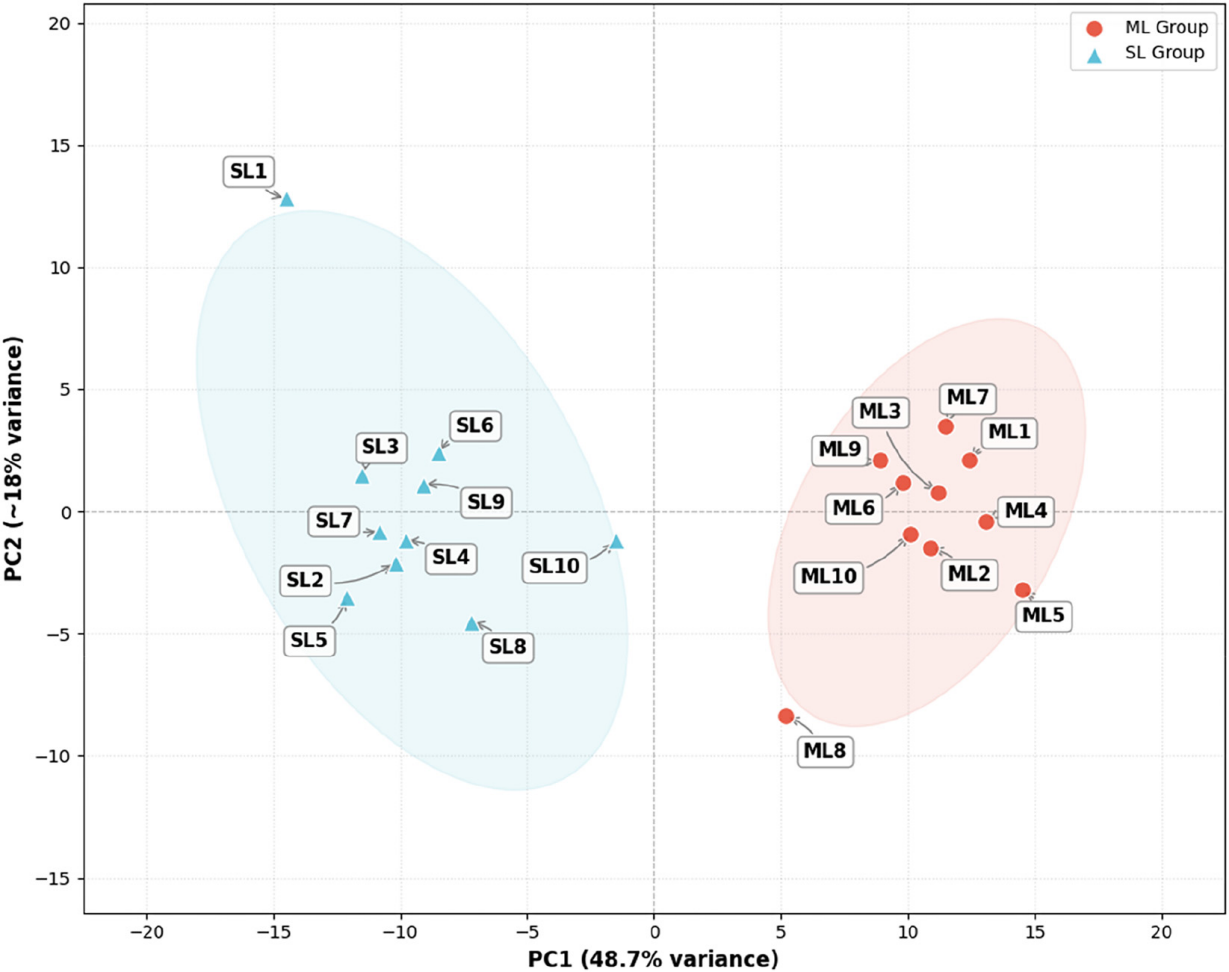
Principal component analysis (PCA) of the 20 samples.

Complementing the correlation data, Principal Component Analysis (PCA) was performed to visualize the global expression patterns among the 20 ovarian samples (Figure 2). The PCA plot demonstrated a separation trend between the ML and SL groups along the first principal component (PC1), which accounted for 48.7 % of the total variance. This indicates that fecundity phenotype is a primary driver of transcriptional variation. While individual variation exists, the robust clustering of the majority of samples validates the experimental design and provides a reliable basis for subsequent differential expression analysis.

### 3.3 CircRNA identification and variable shear (AS) results

**Figure 3:**
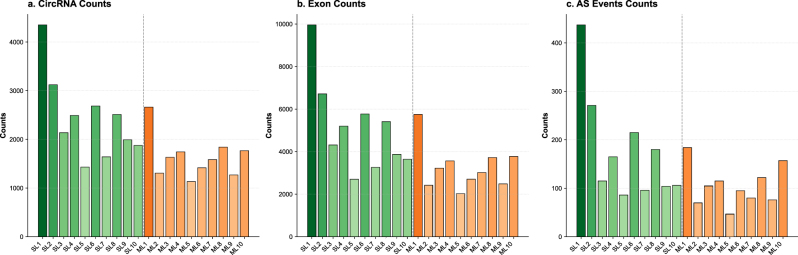
The number of circRNAs, the number of exons, and the number of alternative splicing events in each group were plotted.

A total of 28,451 unique circRNAs were identified across all 20 ovarian samples, derived from 9,874 host genes. Substantial inter-individual variation in circRNA biogenesis and splicing complexity was observed (Table 3, Figure 3). The single-lamb (SL) group exhibited significantly higher circRNA numbers (mean 3,412 ± 682 per sample) and alternative splicing events (mean 298 ± 104 AS events per sample) compared to the multiple-lamb (ML) group (mean 1,489 ± 312 circRNAs and 68 ± 21 AS events per sample; Wilcoxon rank-sum test, *p* < 0.001 for both metrics). The highest circRNA diversity was observed in sample SL1 (4,352 circRNAs, 437 AS events), whereas the lowest was in ML5 (1,133 circRNAs, 47 AS events). The unexpectedly higher circRNA abundance and splicing complexity in the lower-fecundity SL group suggest that circRNAs may primarily function as negative regulators or fine-tuners of fecundity-related pathways in Tibetan sheep ovaries. This increased diversity could reflect a compensatory mechanism or dysregulated back-splicing activity associated with reduced ovulation rate and litter size, consistent with emerging evidence that excessive circRNA production can sequester critical RBPs or miRNAs required for follicular maturation [40].

**Table 3:** A subset of host genes for differentially expressed circRNAs.

circRNA_ID	trancript_IDs	gene_IDs
NC_019459.2:2733923|2741250	XM_004003970.3,XM_012111950.2,XM_015092954.1	101122758
NC_019484.2:107937134|107939165	XM_012107407.1	101120809
NC_019461.2:46217185|46240799	XM_004007835.3,XM_012176675.2,XM_012176676.2,XM_012176677.2,XM_012176678.2,XM_012176679.2,XM_015095088.1	101121766
NC_019468.2:41691301|41691781	XM_004012920.3	101122039
NC_019472.2:65002903|65006958	XM_004016409.3	101106878
NC_019458.2:200401296|200405764	XM_004003081.3,XM_004003082.3,XM_012097855.2,XM_015092512.1,XM_015092513.1,XM_015092514.1,XM_015092515.1,XM_015092516.1	101118404

### 3.4 Cluster analysis of circRNA samples

Differential expression analysis was performed on 10 high-fecundity multiple-lamb (ML) and 10 low-fecundity single-lamb (SL) Tibetan ewes. A total of 82 circRNAs were identified as differentially expressed (*|log_2FC|*≥*1*, *FDR* < *0.05*).

**Figure 4:**
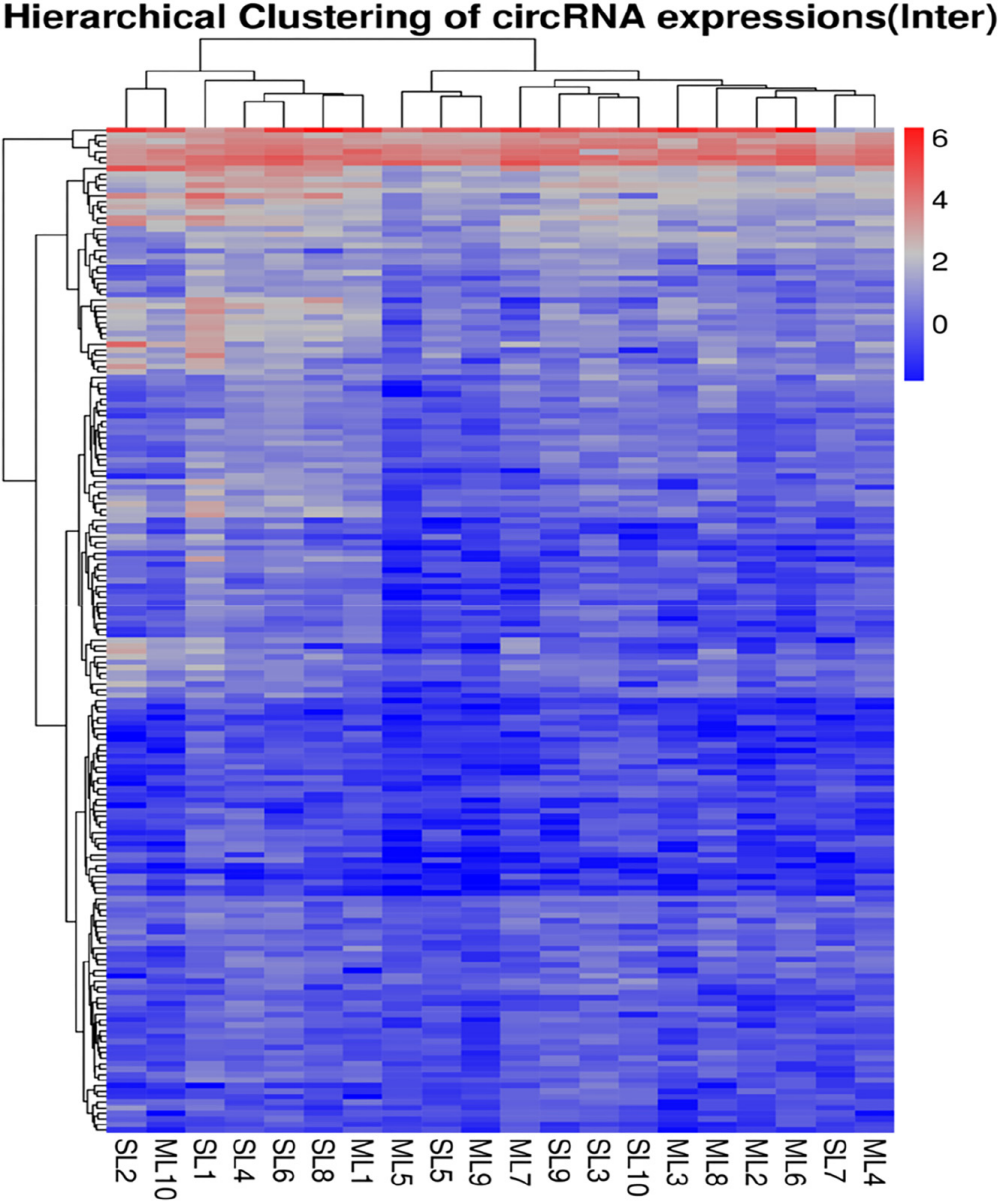
Hierarchical clustering of circRNA expressions.

The hierarchical clustering heatmap of these 82 DECs revealed a complex expression landscape across the 20 samples (Figure 4). Unlike the global trends observed in PCA, the clustering of these specific DECs showed a higher degree of individual variation, with samples from the ML and SL groups appearing interspersed within the dendrogram^13^. While specific gene clusters exhibited differential expression trends (upregulated or downregulated) consistent with the phenotypic groups^14^, the lack of distinct, non-overlapping phenotypic clusters suggests significant biological heterogeneity. This pattern indicates that while these 82 circRNAs are statistically significant markers, their regulatory roles in the Tibetan sheep ovary may function through fine-tuned modulation rather than distinct on/off switching, reflecting the complexity of reproductive traits.

### 3.5 Screening of CircRNA differentially expressed genes

In this study, 82 differentially expressed circRNAs were identified between the monotocous and multiparous polytocous groups, with 35 upregulated and 47 downregulated circRNAs. These ovarian circRNAs exhibited significant expression differences between the two groups, suggesting their potential key roles in ovarian development and fertility regulation. The detailed list of differentially expressed circRNAs is provided in Supplementary Table 2.

**Figure 5:**
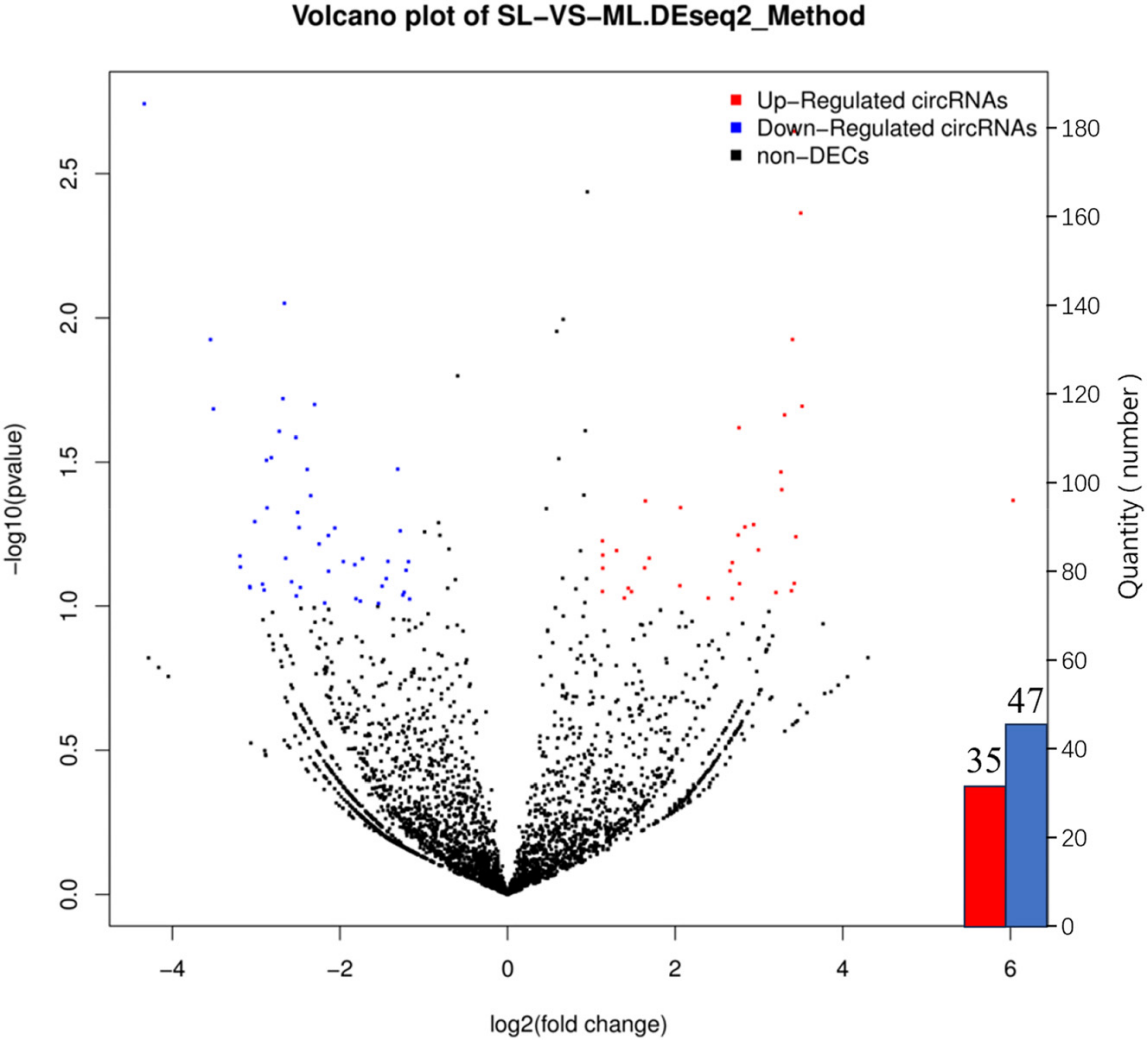
Volcano plot of differentially expressed circRNAs. The *X*-axis represents log_2_-transformed mean expression levels, while the *Y*-axis indicates log_2_ fold change (log_2_FC). Red dots denote upregulated differentially expressed circRNAs (log_2_FC > 0, adjusted *p* < 0. 1), blue dots represent downregulated DECs (log_2_FC < 0, adjusted *p* < 0. 1), and black dots correspond to non-DECs (not statistically significant).

Figure 5 shows the statistics of differentially expressed circRNAs. Figure 5’s volcano plot displays the distribution of 35 upregulated circRNAs (red dots, *log*_*2*_ *FC* > *1*, *p < 0.1*) and 47 downregulated circRNAs (blue dots, *log*_*2*_ *FC < -1*, *p < 0.1*). Gray dots represent non-differentially expressed circRNAs, indicating no significant expression differences between groups.

To highlight the most functionally significant candidates among the 82 differentially expressed circRNAs DE circRNAs we performed a rigorous prioritization based on fold change significance and linkage to known reproduction genes. We predict that the top candidate DE circRNAs listed in Table 4 represent molecules with pivotal regulatory potential in the Tibetan sheep ovary.

**Table 4:** The most influential top 10 DE circRNAs.

circRNA ID	Direction	Fold change	*p* value	Host gene/associated reproduction gene
NC_019471.2:43812560|43813830	Up	3.497979932	0.0043335	LSM14A
NC_019462.2:43091865|43096948	Up	3.424417554	0.0022527	TCF7
NC_019460.2:216082209|216086758	Up	3.305010384	0.0217336	EP300
NC_019459.2:242217795|242219856	Up	2.767920738	0.0835832	E2F2
NC_019464.2:48212845|48216051	Up	1.644204525	0.0431063	RNF111
NC_019458.2:233343283|233354436	Down	−1.169361898	0.0945827	CDC42
NC_019458.2:176469280|176474355	Down	−1.310438699	0.0334299	PAP1B
NC_019470.2:59737796|59761429	Down	−1.730604951	0.0684042	CSNK2A1
NC_019459.2:117036184|117046567	Down	−2.526161495	0.0259672	MAP3K2
NC_019461.2:111287336|111287760	Down	−3.0167877	0.0508721	CUL1

Table 4 presents the top 10 DE circRNAs including their expression direction p values and associated reproduction related host genes or predicted target genes. For example the *circRNA NC 019471.2 43812560|43813830* showed the highest upregulation suggesting a potent regulatory role (*Fold Change 3.497979932*). Conversely *NC 019461.2 111287336|111287760* displayed the most significant downregulation (*Fold Change -3.0167877*). Notably these highly differential circRNAs are linked to genes such as *LSM14A* [41] and *MAP3K2* [42] which are known players in ovarian homeostasis and follicular development.

The volcano plot in Figure 5 shows the distribution of differentially expressed circRNAs, with 35 upregulated circRNAs (red dots, *log*_*2*_ *FC* > *1*, *p < 0.1*) and 47 downregulated circRNAs (blue dots, *log*_*2*_ *FC < -1*, *p < 0.1*). Gray dots represent non-differentially expressed circRNAs, indicating no significant expression differences between the groups.

Extreme regions (*log*_*2*_ *FC* > *2* or *log*_*2*_ *FC < −2*) showed significant expression changes in circRNAs, suggesting that these circRNAs may play key roles in shaping reproductive traits in Tibetan sheep. Most differentially expressed circRNAs (*|log*_*2*_ *FC| = 1–2*) showed moderate changes, reflecting stable regulatory dynamics. In the high significance regions (*−log*_*10*_ *[p]* > *0.5*), circRNAs exhibited pronounced expression dysregulation, indicating their potential as fertility regulators. The volcano plot further validated significant circRNA expression divergence between groups.

### 3.6 GO and KEGG functional analysis of host genes of differentially expressed circRNAs

**Figure 6:**
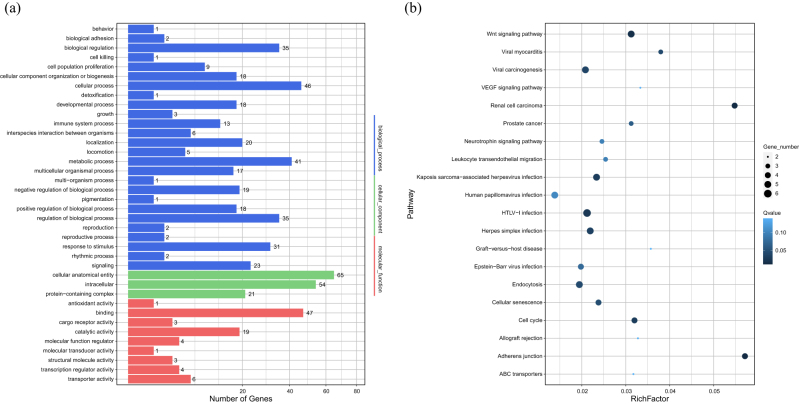
GO and KEGG enrichment maps of differentially expressed circRNA host genes.

Gene Ontology (GO) enrichment analysis was performed on the host genes of the 412 differentially expressed circRNAs (DECs). A total of 38 GO terms were significantly enriched (*Q < 1*) and are presented in order of decreasing relevance to ovarian function and reproduction (Figure 6a).

Reproductive process (BP, 38 genes, *Q* = *0.0018*) ranked highest, centrally involved in folliculogenesis, ovulation, and steroidogenesis. This term indicates that DECs are key determinants of litter size variation, particularly in sheep with year-round estrus. The underlying hormonal regulation is strongly supported by the enrichment of Regulation of hormone metabolic process (BP, 21 genes, *Q* = *0.0027*) and Steroid metabolic process (BP, 19 genes, *Q* = *0.0085*), which collectively regulate the estradiol–progesterone balance crucial for multiple ovulation.

At the molecular level, the top enriched term was Transcription coregulator activity (MF, 18 genes, *Q* = *0.0034*), directly linking circRNAs to the enhanced expression of fecundity-associated gene networks. Furthermore, the significant enrichment of Nuclear speck (CC, 22 genes, *Q* = *0.0093*) suggests circRNA control over pre-mRNA splicing essential for follicle maturation.

The functional response to these hormonal and molecular changes is reflected by Response to steroid hormone (BP, 25 genes, *Q* = *0.0142*) and Follicle development (BP, 15 genes, *Q* = *0.0289*). We hypothesize that these terms collectively demonstrate a circRNA-dependent mechanism that enhances the sensitivity of ovarian cells to steroids, thereby promoting follicular recruitment and contributing to the increased number of ovulatory follicles in high-fecundity individuals.

A total of 20 significantly enriched KEGG pathways (*Q* value < 0.15) were identified, representing the top pathways with the most pronounced enrichment. These pathways encompass a broad spectrum of biological processes, including cell signaling, cell cycle regulation, intercellular communication, immune-inflammatory responses, and viral infection-related mechanisms. The KEGG enrichment bubble plot (Figure 6b) visualizes three key parameters for each pathway. These parameters include the enrichment factor (Rich Factor), the number of enriched genes, and the statistical significance (*Q* value). In this plot, the *X*-axis indicates the Rich Factor, where a higher value reflects a greater proportion of differentially expressed genes involved in the pathway. The *Y*-axis lists the pathway names. The color gradient represents the *Q* value, with darker colors indicating higher enrichment significance.

Among these pathways, several show strong associations with ovarian function and reproductive regulation. The Adherens junction pathway(4 genes, *Q* = *0.0117*) is the most significantly enriched, exhibiting the highest enrichment factor. This pathway plays a critical role in maintaining the adhesion and structural integrity of granulosa cells in the ovary. Differentially expressed circRNAs may influence this pathway by modulating adhesion molecules or cytoskeletal components, which in turn may affect follicular architecture and disrupt follicle development. The Cell cycle pathway (4 genes, *Q* = *0.0311*) contains enriched circRNA host genes such as *E2F2*, *SMC6*, and *DBF4*, which may be involved in regulating mitosis and apoptosis in follicular cells. These genes likely contribute to determining whether follicles progress through normal development or undergo atresia. The Wnt signaling pathway (5 genes, *Q* = *0.0169*) is closely linked to follicle recruitment, maintenance of ovarian stem cells, and granulosa cell differentiation. The observed enrichment of Wnt ligands suggests that circRNAs may influence the fate of ovarian somatic cells and regulate ovulation and follicular maturation via this pathway [43]. The Cellular senescence pathway (4 genes, *Q* = *0.0473*) contains enriched host genes such as *TP53INP1* and *SENP7*. These genes may be involved in terminating follicular development or regulating cellular activity, which could impact the functional lifespan of follicles [44]. The Endocytosis pathway (5 genes, *Q* = *0.0443*) plays an essential role in material uptake and intracellular trafficking in follicular cells. CircRNAs enriched in this pathway may affect membrane dynamics and the recycling of hormone receptors. The Neurotrophin signaling pathway (3 genes, *Q* = *0.0978*) regulates the hypothalamic-pituitary-ovarian (HPO) axis, particularly the secretion of gonadotropin-releasing hormone (GnRH) and follicle-stimulating hormone (FSH). The involvement of circRNAs in this pathway suggests a potential role in neuroendocrine-ovarian regulation. The Leukocyte transendothelial migration pathway (3 genes, *Q* = *0.0958*) is important for maintaining immune homeostasis in the ovary and for ensuring intercellular connectivity among granulosa cells. CircRNAs may modulate this pathway by affecting immune-related factors, thereby influencing local inflammation and the follicular microenvironment.

In addition, other enriched pathways include allograft rejection, VEGF signaling, and ABC transporter pathways. These pathways are potentially involved in immune modulation, ovarian angiogenesis, and metabolite transport, suggesting that circRNAs may participate in reproductive regulation at multiple biological levels [45].

In summary, the findings indicate that fertility-associated circRNAs are likely involved in regulating key ovarian physiological processes through multiple pathways and mechanisms. These include folliculogenesis, as mediated by cell cycle and cellular senescence pathways; steroidogenesis, as influenced by angiogenesis-related signaling; and additional roles in immune homeostasis, cellular adhesion, and neuroendocrine coordination. These regulatory mechanisms collectively contribute to overall reproductive performance.

It is noteworthy that certain virus-related pathways, such as those associated with renal cell carcinoma, HTLV-I infection, and Epstein–Barr virus (EBV) infection, also exhibited unexpectedly significant enrichment. This observation may partly result from annotation overlap with pathways involved in immune or stress response mechanisms. However, such findings merit further investigation. Future studies may validate these results through integrated analyses of expression dynamics and tissue-specific patterns in reproductive organs.

### 3.7 Protein-protein interaction analysis of genes originating from differentially expressed circRNAs

To systematically comprehend the potential molecular functions of the differentially expressed circRNAs, we constructed Protein-Protein Interaction (PPI) networks based on both the host genes originating from the DE-circRNAs and their predicted microRNA target genes. We utilized the STRING database (v. 11.5) to build these networks, setting the minimum required interaction confidence score to 0.40 (low confidence) to capture a broader range of associations. Subsequently, the networks were visualized and centrality analyses were performed using Cytoscape software (v. 3.10.3), with the core Hub genes defined by the highest Degree values. Network analysis identified the top five Hub genes from both the upregulated and downregulated circRNA-associated networks (see Supplementary Table 3).

**Figure 7:**
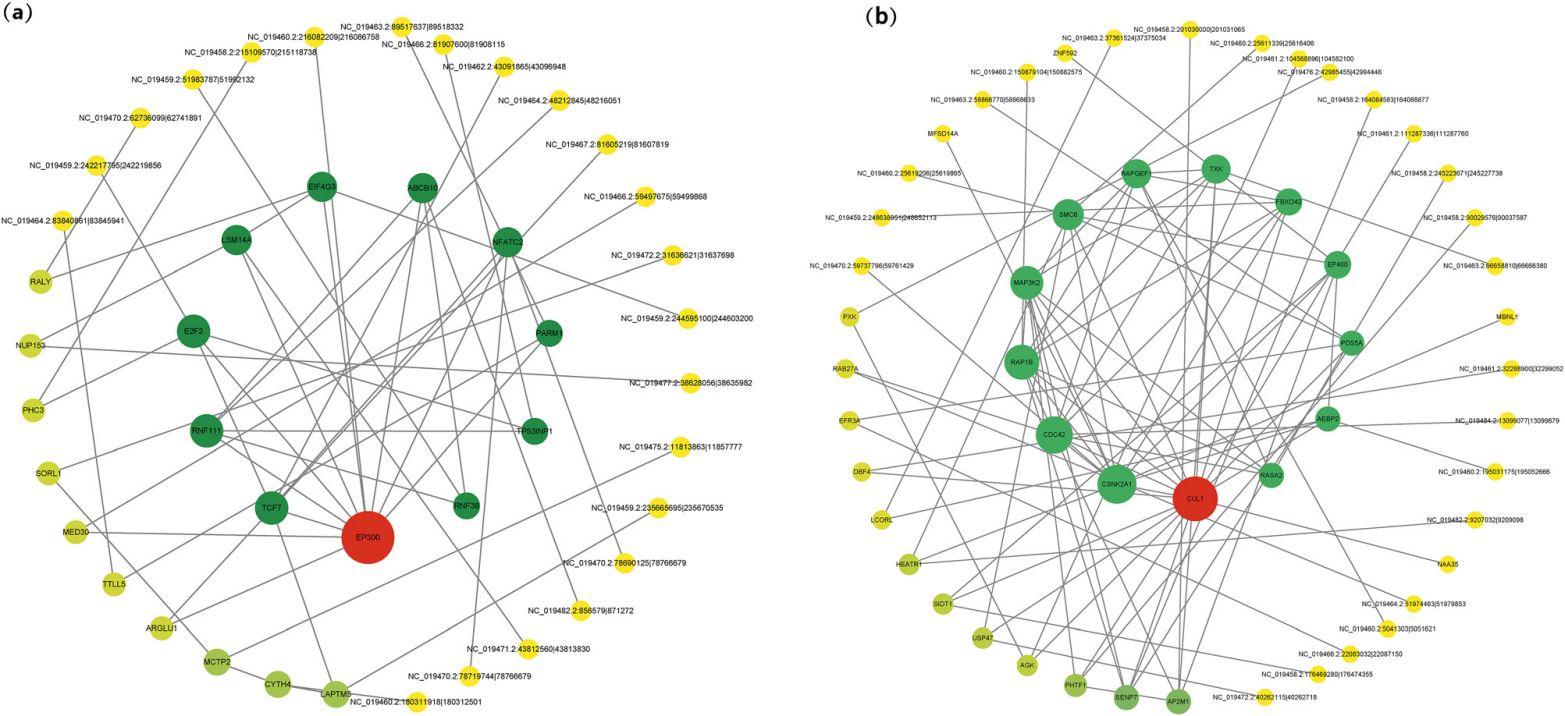
Protein-protein interaction (PPI) network of host genes corresponding to differentially expressed circRNAs.

The upregulated circRNA-associated network (Figure 7a) consisted of 43 nodes and 48 edges. We hypothesize that EP300 acts as a central hub in this network. We suggest that these upregulated circRNAs exert their regulatory effects via two mechanisms, First, by regulating the expression of their host genes (e.g., direct transcriptional regulation), and second, indirectly by acting as competitive endogenous RNAs (ceRNAs) to influence the expression of predicted miRNA target genes (e.g., *E2F2*).

The upregulation of circRNAs may enhance EP300-mediated histone acetylation and transcriptional co-activation functions, thereby coordinating gene expression with dynamic cellular states [46]. Concurrent upregulation of immune or antiviral-related nodes such as *USP18* and *NFATC2* suggests that cells might be in an activated interferon or stress response state. The upregulation of transcription factors including *TCF7* and *E2F2*, and their close interactions with *EP300*, indicates enhanced proliferative or transcriptional activity [47], 48]. Additionally, the upregulation of *CYTH4* may promote membrane vesicle dynamics. Collectively, these consistent upregulation patterns suggest that differential circRNAs may drive a series of synchronized regulatory events that promote active proliferation and metabolism in follicular cells. In contrast, the downregulated circRNA-associated network (Figure 7b) was larger, composed of 53 nodes and 85 edges. Unlike the upregulated network, the primary core node in this network is *CUL1*. As a core component of the ubiquitin ligase complex, we speculate that a decrease in the expression of circRNAs linked to *CUL1* may indirectly lead to the suppression of *CUL1*-mediated protein degradation, cell cycle regulation, and cytoskeletal remodeling [49], [50], [51]. This inhibitory effect predicts a reduction in protein homeostasis and signal transduction efficiency.

Downregulation of genome maintenance factors such as *PDS5A* and *SMC6* implies weakened DNA repair and sister chromatid separation capacity, which could pose a potential obstacle to the rapid cell division required for high fecundity. Furthermore, the downregulation of vesicle transport factors, including *RAB27A* and *AP2M1*, suggests decreased receptor endocytosis or secretion functions, which could impair the ovarian cell response to external signals like gonadotropins. These observed downregulation trends suggest that impaired protein homeostasis and genomic instability may be associated with certain low-fecundity phenotypes.

Network Quantification and Regulatory Mode, The percentage of shared genes between the two networks is relatively low (approximately 5–7% shared nodes), which suggests that the upregulated and downregulated circRNAs may function through relatively independent and antagonistic pathways. We propose that differential circRNAs influence these central Hub nodes within the PPI network, thereby generating multi-level synergistic or antagonistic effects. These effects mediate complex cellular functional changes at biological layers including transcriptional regulation, immune/stress responses, proliferation initiation, membrane and vesicle processes, protein homeostasis, and genome maintenance, ultimately impacting the fertility of Tibetan sheep.

Overall, the PPI network analysis indicates that differentially expressed circRNAs may exert multi-layered regulatory effects by modulating central hub genes. This could potentially reshape transcriptional programmes, immune responses and cellular homeostasis in a coordinated manner.

### 3.8 Targeted prediction of results

The target prediction strategy prioritized confidence and specificity. Algorithm Selection and Differences The selection of TargetScan miRanda PITA and IntaRNA reflects diverse mechanistic models. TargetScan and miRanda focus on seed matching and overall binding stability. PITA distinctively evaluates target site accessibility energy using a double Delta G metric essential for effective binding dynamics. IntaRNA specializes in general RNA interaction thermodynamics. This varied approach ensures comprehensive yet complementary prediction results. Intersection Justification and Thresholds We used an intersection approach to significantly reduce the high false positive rates inherent in single algorithm predictions thereby improving specificity. The strength lies in establishing a high confidence candidate set. The limitation is potential exclusion of biologically relevant targets showing low algorithmic consensus. To balance this for animal circRNA miRNA pairs a minimum consensus of two out of three tools was required. The triple intersection was used for defining the highest confidence core networks presented in Figure 10 to maximize statistical rigor for validation. Prediction Parameters Specific parameters enhanced prediction stringency. For miRanda we mandated a minimum score of 140 and an energy cutoff of 5 kcal mol. PITA utilized a double Delta G context cutoff of 70. Other tools TargetScan IntaRNA psRobot and TargetFinder were run using their default settings.

**Figure 8:**
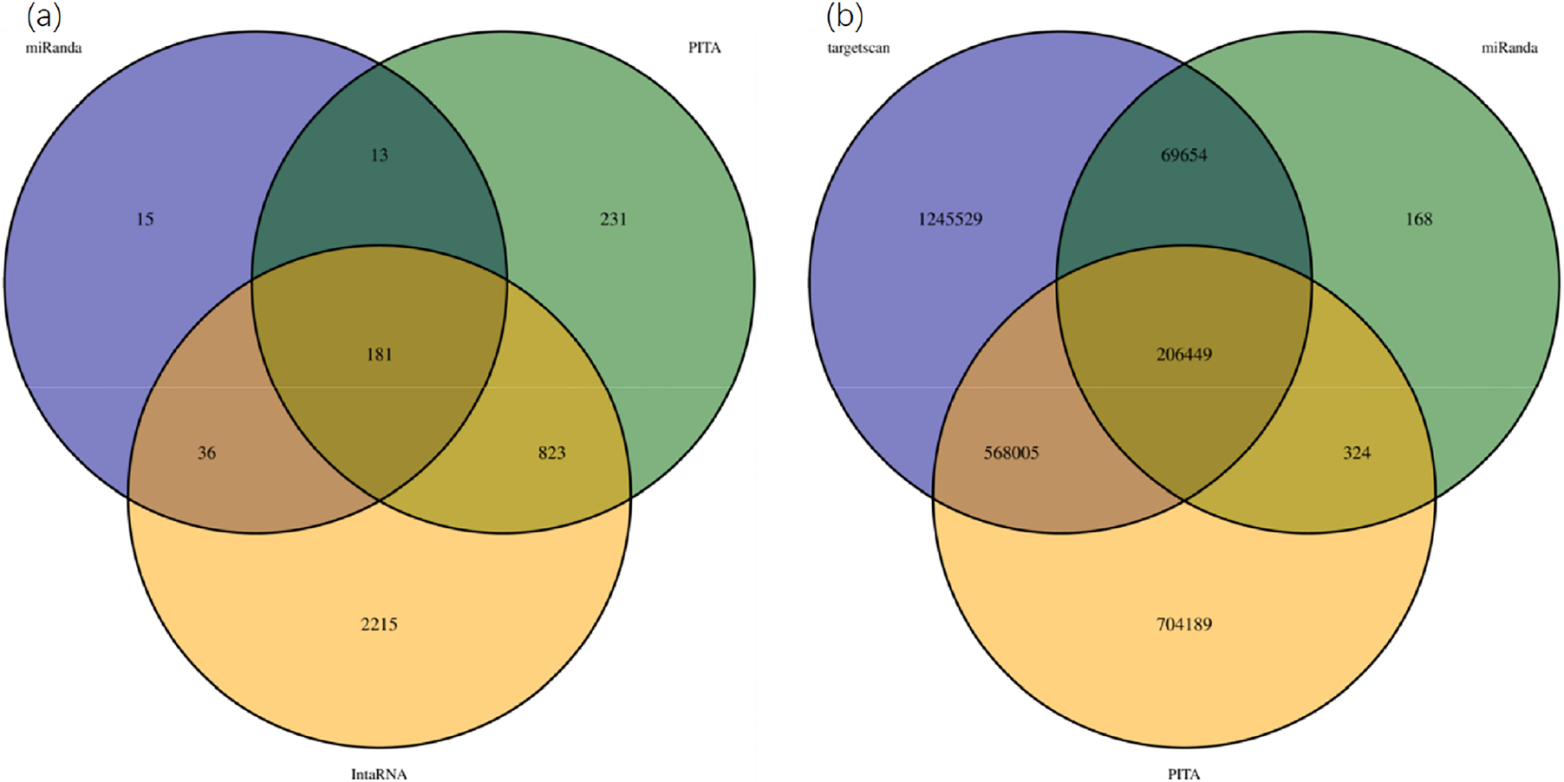
Venn plots of target genes of circRNAs predicted by various algorithms.

As illustrated in Figure 8a, the Venn diagram delineates the overlap of circRNA-targeting (*n* = 82) miRNAs predicted by three distinct algorithms (miRanda, *PITA*, and IntaRNA), along with their intersection profiles. Algorithm-specific predictions yielded 15 (miRanda), 231 (PITA), and 2,215 (IntaRNA) unique miRNAs, respectively, reflecting substantial methodological divergences in miRNA target prioritization. Pairwise intersections revealed 13 miRNAs shared by miRanda and *PITA*, 36 by miRanda and IntaRNA, and 823 by *PITA* and IntaRNA. Notably, the triple intersection – representing consensus predictions across all three algorithms – comprised 181 high-confidence miRNAs, which are strong candidates for bona fide circRNA-mRNA interaction partners.

The candidate target miRNAs associated with differentially expressed circRNAs (DECs) are systematically cataloged in the following Table 5. Our screening pipeline identified 151 high-confidence miRNAs, 76 putative target genes, and 1,054 miRNA-mRNA regulatory pairs. These robust interaction networks establish a critical foundation for downstream functional enrichment analyses and experimental verification. Notably, the identified miRNAs and their cognate targets are hypothesized to serve as pivotal regulators in key reproductive processes, including ovarian homeostasis modulation, folliculogenesis dynamics, and fertility optimization.

**Table 5:** Prediction results of circRNA-targeted miRNAs using three algorithms (TargetScan, miRanda, PITA).

Software	miRNA_number	Target_gene_number	miRNA:target_number
miRanda	89	46	245
PITA	149	78	1248
IntaRNA	154	83	3255
Result	151	76	1054

Figure 8b presents the intersection and algorithm-specific components of miRNA-target predictions generated by TargetScan, miRanda, and PITA. TargetScan yielded the most extensive prediction scope with 1,245,529 miRNA-mRNA regulatory pairs, followed by PITA (704,189 pairs), while miRanda exhibited stringent prediction criteria (168 pairs). The tripartite consensus predictions across all three algorithms identified 206,449 high-confidence regulatory pairs, representing the highest-confidence predictions. Pairwise intersections revealed 69,654 shared pairs between miRanda and TargetScan, 324 between miRanda and PITA, and 568,005 between TargetScan and PITA. These substantial discrepancies in prediction outputs reveal critical algorithmic divergence in miRNA target prediction methodologies.

As shown in the Table 6, three miRNA target prediction tools (TargetScan, miRanda, and *PITA*) identified approximately 41,000 to 43,000 potential miRNA target genes, with predicted miRNA-mRNA pairing relationships ranging from 270,000 pairs (miRanda) to over 2 million pairs (TargetScan). Considering differences in prediction algorithms and accuracy among these tools, this study employed an intersection strategy to select 42,703 potential miRNA target genes and 844,432 high-confidence miRNA-mRNA regulatory pairs. These results were used for subsequent construction of the ceRNA regulatory network and functional enrichment analysis.

**Table 6:** Prediction results of miRNA-targeted mRNAs using three algorithms (TargetScan, miRanda, PITA).

Software	miRNA_number	Target_gene_number	miRNA:target_number	Target_location_number
Targetscan	150	42985	2089637	3201050
miRanda	150	40981	276595	319241
PITA	150	42983	1478967	15530238
Result	150	42703	844432	–

**Figure 9:**
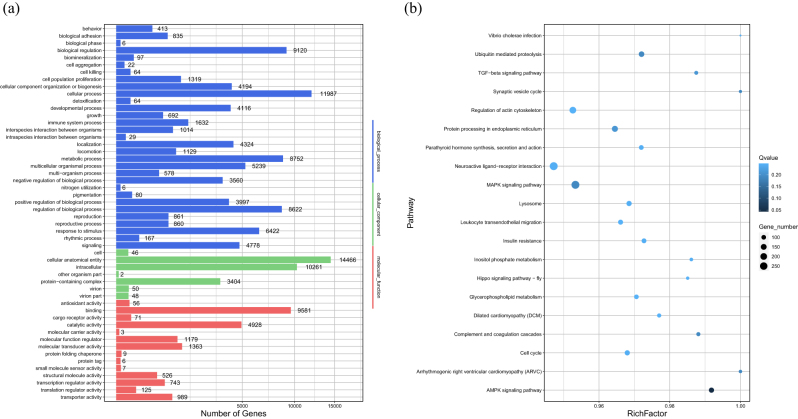
GO and KEGG enrichment maps of miRNA target genes.

As shown in Figure 9a, we hypothesize that the most biologically relevant enrichments center on processes directly governing ovarian function and signaling. Enrichment was highly significant in cell population proliferation (1,319 genes) and immune system processes (1,632 genes) indicating important roles in follicular growth and reproductive immune regulation. Furthermore the key molecular functions driving these specific processes included receptor activity (71 genes) and transporter activity (989 genes) revealing roles in cellular signal transduction and material transport vital for ovulation dynamics. These functions are closely related to ovarian development follicular growth and ovulation potentially affecting the reproductive capacity of Tibetan sheep.

We predict that the underlying regulation is built upon fundamental and comprehensive cellular activities. The most highly enriched biological processes were cellular processes (11,987 genes, *Q* = 0.0082) biological regulation (9,120 genes, *Q* = 0.0247) and metabolic processes (8,752 genes, *Q* = 0.0385) demonstrating broad control over energy homeostasis and cellular survival. The primary molecular mechanisms were binding activity (9,581 genes, *Q* = 0.0397) and catalytic activity (4,928 genes, *Q* = 0.0452) suggesting widespread protein interactions and enzymatic activity. The target genes were primarily distributed in cells (14,466 genes, *Q* = 0.0196) intracellular regions (10,261 genes, *Q* = 0.0213) and protein containing complexes (3,404 genes, *Q* = 0.0498). This localization suggests that miRNA target genes are involved in regulating cellular structure and protein assembly essential for follicular cell homeostasis and overall ovarian function.

GO enrichment analysis strongly suggests that miRNA target genes widely participate in critical functions such as immune regulation protein interactions and signal transduction directly influencing ovarian development steroid synthesis and reproduction related signaling pathways. We predict these results provide a comprehensive theoretical basis for in depth exploration of miRNA mediated molecular networks underlying reproductive traits in Tibetan sheep.

As shown in Figure 9b, the KEGG enrichment bubble plot of miRNA target genes reveals significant enrichment of several key biological pathways, suggesting their potential involvement in ovarian function regulation. We predict that energy metabolism is a core regulated function due to the highly significant enrichment of the AMPK signaling pathway. AMPK signaling is central to cellular energy sensing and reproductive function. Key miRNA target genes associated with this pathway include *SLC25A26* and *USP47* which are also circRNA source genes. *SLC25A26* regulates mitochondrial SAM transport [52] and is linked to premature ovarian failure. *USP47* is involved in deubiquitination processes and may contribute to energy metabolism and cell cycle progression [53]. Their involvement suggests that the circRNA miRNA axis affects ovarian function by maintaining metabolic homeostasis. Other related pathways such as Insulin Resistance involving genes like *ABCB10* and *TXK* further underscore metabolic control.

We hypothesize that the network plays a direct role in maintaining ovarian integrity through immune and stress responses. Pathways such as the Complement and Coagulation Cascades involving *PDLIM2* and *LAPTM5* may modulate local immune responses in ovarian tissue. Protein processing in the endoplasmic reticulum involving *AGK* and *EIF4G3* regulates stress responses and protein folding. Ubiquitin mediated proteolysis with genes like *RNF38* and *SENP7* maintains protein homeostasis affecting overall follicular health. We speculate that enriched disease related pathways like *Vibrio cholerae infection* are related to overlapping annotations involving these immune or stress response mechanisms.

We predict that direct signal transduction pathways are pivotal regulators of follicular dynamics and hormone synthesis. The MAPK signaling pathway known to regulate granulosa cell proliferation and steroidogenesis involves target genes like *RAP1B MAP3K2* and *EP300* which mediate MAPK activation ERK signaling and transcriptional coactivation respectively. Additionally TGF beta signaling involving *SMC6 USP18* and *MCTP2* regulates chromatin stability and follicular development. Pathways crucial for neuroendocrine signaling such as Neuroactive Ligand Receptor Interaction involving *GPRC5C* and *NFATC2* and the Hippo signaling pathway involving *EP400 FBXO42* and *TCF7* contribute significantly to follicle fate regulation.

We confirm that the enrichment of the Cell Cycle pathway with targets like *TP53INP1* [54] *CDC42* and *DBF4* directly highlights the network’s role in mitosis DNA replication and stress responses crucial for cellular division during folliculogenesis. These findings collectively underscore the role of the identified pathways in follicular development and hormone synthesis providing a strong basis for experimental verification.

**Figure 10:**
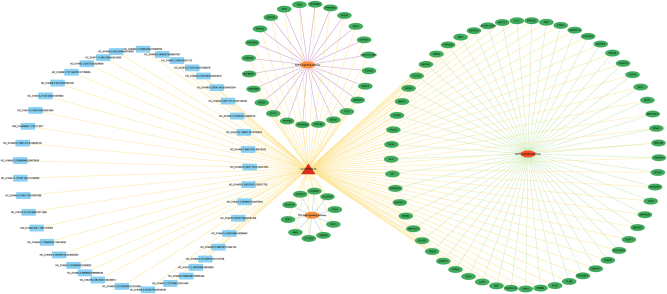
CircRNA–miRNA–mRNA–pathway regulatory network.

As shown in the Figure 10, based on differential expression analysis and target gene prediction, a ceRNA network centered on the differentially expressed miRNA *oar-miR-654-5p* was constructed in this study. This network includes upstream circRNAs on the left, the central miRNA node, target mRNAs, and their associated functional pathways. Analysis revealed that 41 circRNAs contain potential binding sites for *oar-miR-654-5p*. Notably, circRNAs such as *NC_019458.2:106614774|106638100* and *NC_019459.2:244595100|244603200* were predicted to have strong binding affinities. These circRNAs likely act as competing endogenous RNAs (ceRNAs), sequestering miRNAs through a sponge effect, thereby modulating the miRNA-mediated negative regulation of downstream target genes. Previous studies have indicated that circRNAs in mammalian ovaries can regulate reproduction-related gene expression by modulating miRNAs and constitute an important component of ceRNA regulatory networks [55].

The target genes of *oar-miR-654-5p* encompass several key factors related to reproduction and energy metabolism. For example, *PPARG* is an important regulator of cell proliferation and differentiation and has been shown to directly promote proliferation in ovine granulosa cells [56]. *FOXO3* is a key gene involved in the maintenance and activation of primordial follicles and has been confirmed across multiple mammalian species to play an irreplaceable role in ovarian reserve regulation [57]. Additionally, *MAP3K14* and *SMAD9* participate in the MAPK and TGF-β signaling pathways, respectively, regulating cellular inflammatory responses and follicular differentiation, which are crucial for maintaining ovarian microenvironment stability and follicle growth [58], [59], [60].

KEGG enrichment analysis revealed that these target genes were significantly enriched in three classical signaling pathways closely related to ovarian function. The AMPK signaling pathway regulates follicle growth by modulating cellular energy balance and controls steroidogenesis and germ cell maturation in ovarian cells, including granulosa cells, theca cells, and luteal cells [61]. Genes such as *PPARG*, *SCD*, and *ADIPOQ* synergistically regulate this pathway. The MAPK signaling pathway plays a vital role in follicle growth, ovulation induction, and ovarian inflammatory responses, with core kinases like *MAP3K14* and *MAP4K4* belonging to this pathway. The TGF-β signaling pathway maintains ovarian functional homeostasis by regulating granulosa cell proliferation and luteal formation, with key components identified in this study including *SMAD9*, *ACVR1C*, and *AMH* [60]. Collectively, these results suggest that *oar-miR-654-5p* may function as a key miRNA, regulating the expression of reproduction-related genes through interactions with specific circRNAs, thereby influencing the activity of multiple critical signaling pathways in ovine ovarian tissue.

## 4 Discussion

This study represents the first systematic comparison of circular RNA circRNA expression profiles in the ovarian tissue of high and low fecundity Tibetan sheep using high throughput sequencing. We identified a total of 82 differentially expressed circRNAs DE circRNAs with 35 upregulated and 47 downregulated providing a crucial molecular foundation for understanding the reproductive physiology of this plateau adapted breed.

### 4.1 Comparative analysis and biological context

Compared to other ovine studies the total number of circRNAs identified here approximately 2030 is relatively conservative. This might be attributed to the sequencing depth bioinformatics filtering criteria employed or the unique reproductive regulatory mechanisms inherent to Tibetan sheep in extreme high altitude environments. Prior reports in other breeds such as Small tailed Han sheep have documented over 21000 circRNAs highlighting that variations in circRNA numbers underscore the species specificity and environmental adaptability of fecundity regulation.

Liu et al.’s study on Hampshire sheep [62] identified 4256 circRNAs with 183 significantly differentially expressed primarily enriched in EGF EGFR RAS JNK TGF beta and thyroid hormone signaling pathways. Similarly Wang et al. [63] found 21531 and 20572 circRNAs in Cele Black and Hetian sheep ovaries respectively. Host genes of those DE circRNAs were significantly enriched in MAPK and Hippo signaling pathways critical for follicular development. These findings show high concordance with our observed enrichment of multiple reproduction related pathways suggesting conserved regulatory roles of circRNAs across sheep breeds in pathways essential for follicular maturation and hormone synthesis [55].

The host genes of our identified DE circRNAs were primarily associated with ovarian function follicular maturation and steroid metabolism aligning with findings in domestic livestock. For instance Liu et al.’s goat study [64] and Jia et al.’s porcine study [43] confirmed that DE circRNAs and their host genes are enriched in PI3K Akt MAPK Notch and FOXO signaling pathways all tightly linked to follicular development hormone synthesis and oocyte maturation. Additionally Li et al. [40] observed significant circRNA expression differences between large and small follicles suggesting stage specific expression patterns during follicular development.

### 4.2 Functional elucidation and mechanistic insights

Gene Ontology GO and KEGG enrichment analyses further elucidated the potential functions of the DE circRNAs in reproductive processes. The enrichment results prominently pointed towards cell growth cell cycle and energy metabolism which are known rate limiting steps for follicular development and oocyte maturation.

Notably the AMPK signaling pathway was highly enriched in this study. Given its role as a cellular energy sensor this pathway is central to maintaining ovarian homeostasis and regulating ovulation in response to metabolic signals [65]. This suggests that the high fecundity phenotype in Tibetan sheep may be intricately linked to enhanced local ovarian energy metabolic efficiency. Liu et al. reported that key genes in these pathways are highly expressed in high fertility goat ovaries and are tightly associated with follicular survival and ovulatory capacity [66].

Other enriched pathways such as TGF beta signaling [67] MAPK signaling and Cell Cycle play essential roles in regulating ovarian cellular structural integrity folliculogenesis and hormone biosynthesis. Protein Interaction PPI network analysis identified several core target genes including *PPARG FOXO3* and *SMAD9* all of which have established biological roles in ovarian function apoptosis and reproductive endocrine regulation thus providing a basis for subsequent mechanistic investigations.

### 4.3 CircRNA regulatory axis and biological significance

The most significant novelty of this study lies in the first ever construction of a circRNA based competitive endogenous RNA ceRNA regulatory network in Tibetan sheep. Our analysis unequivocally identified oar-mir-654-5p as a novel hub microRNA which competitively binds to key DE circRNAs such as *NC 019459.2 244595100|244603200* consequently releasing and upregulating downstream target genes like *PPARG* and *FOXO3*.

This finding is significantly strengthened by the observed overlap between circRNA host gene pathways and miRNA target gene pathways. We hypothesize this convergence suggests a coordinated regulatory mechanism where the circRNA acts as a functional component of the pathway architecture validating the predicted ceRNA network structure.

The downstream targets of oar-mir-654-5p include several key genes closely related to ovarian function such as *PPARG FOXO3 ADIPOQ MAP3K14* and *SMAD9*. *PPARG* and *ADIPOQ* in the AMPK pathway regulate energy metabolism and lipid homeostasis influencing follicular development activity and oocyte quality [56]. *FOXO3* serves as a key factor in maintaining the dormancy of primordial follicles and its dysregulated expression may lead to ovarian reserve dysfunction [68]. Kinases such as *MAP3K14 MAP4K4* and *TAOK1* in the MAPK pathway are closely associated with granulosa cell proliferation lipid synthesis and progesterone secretion [68]. Additionally genes in the TGF beta pathway including *SMAD9 ACVR1C* and *AMH* participate in granulosa cell differentiation follicle recruitment and luteal function regulation [69].

The identification of this quaternary regulatory axis circRNA oar-mir-654-5p mRNA AMPK Pathway not only fills a knowledge gap regarding circRNA involvement in Tibetan sheep fecundity but also provides a molecular mechanism specific to the high fecundity phenotype of this plateau adapted breed.

### 4.4 Limitations and critical evaluation

This study primarily relied on high throughput sequencing and in silico prediction to construct the regulatory network. Consequently a major limitation is the lack of *in vivo* or *in vitro* molecular experimental validation to confirm the direct interactions among the circRNA miRNA and mRNA components. Although existing studies demonstrate that circRNAs regulate granulosa cell proliferation via interaction with key miRNAs [55] our predicted interactions require verification. Furthermore ovarian tissue exhibits significant tissue heterogeneity the sequencing results represent an average expression across multiple cell types oocytes granulosa and theca cells. Future work should utilize techniques such as laser capture microdissection for precise analysis of specific cell types. Finally the disparity in circRNA numbers compared to other reports highlights the need for a more comprehensive investigation into circRNA biogenesis and annotation in Tibetan sheep.

### 4.5 Practical applications and future outlook

The findings of this study possess significant translational value. The identified DE circRNAs and oar-mir-654-5p could serve as potential molecular genetic markers for assisted selection and molecular breeding programs in Tibetan sheep to enhance their prolificacy. To translate these results into practice we propose the following experimental strategies:

(1) Functional Validation Use the Dual Luciferase Reporter Assay to confirm the direct binding relationship between the circRNA miRNA and mRNA components.
(2) Mechanistic Exploration Employ siRNA mediated gene knockdown or overexpression experiments in granulosa cells or oocytes to assess the impact of core circRNAs on cell proliferation and apoptosis.
(3) Environmental Influences Future studies should also account for the influence of environmental or physiological factors on circRNA expression such as high altitude adaptation seasonal changes or nutritional status to fully understand their potential impact on ovarian function and fertility in Tibetan sheep.

## 5 Conclusions

This study employed high-throughput sequencing to systematically analyze the circRNA expression profile in Tibetan sheep ovaries, successfully identifying 82 differentially expressed circRNAs between high- and low-fecundity groups. Functional enrichment analyses indicate that these DE-circRNAs and their host genes are primarily involved in crucial biological processes, including cell proliferation, energy metabolism, and immune regulation, with significant enrichment in critical regulatory cascades such as the AMPK signaling pathway and Cell cycle.

The foremost contribution of this research is the construction of a circRNA-miRNA-mRNA competitive endogenous RNA (ceRNA) regulatory network. This integrated analysis identified oar-miR-654-5p as a central regulatory hub within this network, mediating the cross-talk between DE-circRNAs and downstream key target genes (e.g., *PPARG* and *FOXO3*). This finding offers novel insights into the multi-level molecular regulation governing the reproductive performance of plateau-adapted Tibetan sheep.

In summary, this study not only pioneers the understanding of circRNA’s role in Tibetan sheep fertility variation but also provides promising candidates for molecular genetic biomarkers to guide molecular breeding strategies and enhance reproductive efficiency. To translate these *in silico* findings into biological knowledge, further rigorous experimental validation and functional studies of the identified ceRNA axis, particularly the oar-miR-654-5p mediated regulatory loop, are warranted.

## Supplementary Material

Supplementary Material
