# Supplementary material for: Differential expression and functional analysis of circular RNA in ovaries of Tibetan sheep with different fecundity
Source: Open Life Sci. 2026 May 4;21(1):20251256. doi: 10.1515/biol-2025-1256 (PMC13135677; doi:10.1515/biol-2025-1256)
Supplement: Supplementary file 1 — Supplementary Material [file j_biol-2025-1256_suppl_001.docx]

**Supplementary Table**

**Supplementary Table 1:** RNA quality parameters of ovarian samples from 20 Tibetan sheep.

| **Sample ID** | **Group** | **RIN** | **28S/**  **18S** | **Concentration (ng/μL)** | **Total amount (μg)** | **A260/**  **A280** | **A260/**  **A230** |
| --- | --- | --- | --- | --- | --- | --- | --- |
| SL01 | SL | 9.3 | 1.5 | 68 | 3.40 | 2.08 | 2.21 |
| SL02 | SL | 9.1 | 1.3 | 62 | 3.10 | 2.10 | 2.18 |
| SL03 | SL | 9.4 | 1.6 | 78 | 3.90 | 2.09 | 2.24 |
| SL04 | SL | 8.9 | 1.2 | 64 | 3.20 | 2.07 | 2.16 |
| SL05 | SL | 9.5 | 1.6 | 72 | 3.60 | 2.11 | 2.23 |
| SL06 | SL | 9.0 | 1.3 | 66 | 3.30 | 2.08 | 2.19 |
| SL07 | SL | 9.2 | 1.4 | 70 | 3.50 | 2.10 | 2.22 |
| SL08 | SL | 8.8 | 1.1 | 58 | 2.90 | 2.06 | 2.15 |
| SL09 | SL | 9.6 | 1.6 | 80 | 4.00 | 2.12 | 2.25 |
| SL10 | SL | 9.0 | 1.3 | 65 | 3.25 | 2.09 | 2.20 |
| ML01 | ML | 9.4 | 1.5 | 74 | 3.70 | 2.11 | 2.24 |
| ML02 | ML | 9.2 | 1.4 | 69 | 3.45 | 2.10 | 2.21 |
| ML03 | ML | 9.5 | 1.6 | 76 | 3.80 | 2.12 | 2.26 |
| ML04 | ML | 8.7 | 1.1 | 60 | 3.00 | 2.05 | 2.14 |
| ML05 | ML | 9.3 | 1.5 | 71 | 3.55 | 2.09 | 2.22 |
| ML06 | ML | 9.1 | 1.3 | 67 | 3.35 | 2.08 | 2.19 |
| ML07 | ML | 9.0 | 1.4 | 73 | 3.65 | 2.10 | 2.23 |
| ML08 | ML | 9.6 | 1.6 | 82 | 4.10 | 2.13 | 2.27 |
| ML09 | ML | 8.9 | 1.2 | 63 | 3.15 | 2.07 | 2.17 |
| ML10 | ML | 9.4 | 1.5 | 70 | 3.50 | 2.11 | 2.22 |

**Supplementary Table 2:** Complete list of differentially expressed CircRNAs.

| CircRNA_ID | Gene_ID | SL-Expression | ML-Expression | log2FoldChange  (ML/SL) | Pvalue | Padj | Up/Down-Regulation |
| --- | --- | --- | --- | --- | --- | --- | --- |
| NC_019476.2:35215291\|35239510 | 100147801 | 4.21 | 0.91 | -1.80885 | 0.094385 | 0.992011 | Down |
| NC_019461.2:116791198\|116792059 | 101117415 | 0.31 | 2.26 | 3.514302 | 0.020268 | 0.992011 | Up |
| NC_019472.2:40262115\|40262718 | 101122304 | 6.41 | 1.83 | -1.49686 | 0.085289 | 0.992011 | Down |
| NC_019464.2:40260072\|40283730 | 101121015 | 10.19 | 3.25 | -1.18309 | 0.070096 | 0.992011 | Down |
| NC_019460.2:180311918\|180312501 | 101103309 | 2.11 | 3.23 | 2.063738 | 0.045503 | 0.992011 | Up |
| NC_019475.2:18829358\|18829785 | n/a | 0.42 | 3.08 | 3.399026 | 0.011883 | 0.992011 | Up |
| NC_019460.2:180612498\|180614070 | 101103965 | 0 | 1.2 | 3.387428 | 0.088529 | 0.992011 | Up |
| NC_019475.2:11813863\|11857777 | 101119255 | 4.04 | 7.22 | 1.393678 | 0.093844 | 0.992011 | Up |
| NC_019481.2:34496210\|34497840 | n/a | 7.96 | 0.57 | -2.66336 | 0.00889 | 0.992011 | Down |
| NC_019472.2:31636621\|31637698 | 101117962 | 9.96 | 11.41 | 1.135995 | 0.066498 | 0.992011 | Up |
| NC_019482.2:9207032\|9209098 | 101116117 | 4.4 | 0.32 | -2.681 | 0.019075 | 0.992011 | Down |
| NC_019458.2:90029576\|90037587 | 101119349 | 4.62 | 0.35 | -2.7236 | 0.024773 | 0.992011 | Down |
| NC_019469.2:24207841\|24214624 | 101106862 | 5.92 | 0.43 | -2.505 | 0.047239 | 0.992011 | Down |
| NC_019460.2:25611339\|25616406 | 101123020 | 8.07 | 2.36 | -1.82332 | 0.071825 | 0.992011 | Down |
| NC_019477.2:26879643\|26930121 | n/a | 5.81 | 15.8 | 6.031585 | 0.042922 | 0.992011 | Up |
| NC_019470.2:16539620\|16608077 | n/a | 4.96 | 5.7 | 1.132202 | 0.059347 | 0.992011 | Up |
| NC_019460.2:150879104\|150882575 | 101106317 | 17.17 | 4.09 | -1.31044 | 0.03343 | 0.992011 | Down |
| NC_019468.2:55894361\|55895446 | 101105426 | 0.47 | 1.53 | 2.831757 | 0.053098 | 0.992011 | Up |
| NC_019460.2:216082209\|216086758 | 101110136 | 0.27 | 2.09 | 3.30501 | 0.021734 | 0.992011 | Up |
| NC_019460.2:25619206\|25619865 | 101123020 | 24.89 | 6.95 | -1.28087 | 0.054766 | 0.992011 | Down |
| NC_019459.2:242217795\|242219856 | 105606975 | 0.6 | 1.66 | 2.767921 | 0.083583 | 0.992011 | Up |
| NC_019475.2:21501665\|21511066 | 101109304 | 3.3 | 0.36 | -2.52041 | 0.092262 | 0.992011 | Down |
| NC_019458.2:106614774\|106638100 | 101113122 | 2.29 | 7.9 | 3.271779 | 0.039396 | 0.992011 | Up |
| NC_019474.2:44635031\|44640774 | 101117449 | 4.36 | 0.36 | -2.87695 | 0.031127 | 0.992011 | Down |
| NC_019470.2:78690125\|78766679 | 101119239 | 0 | 0.95 | 3.439713 | 0.057403 | 0.992011 | Up |
| NC_019460.2:10225350\|10230281 | 101105732 | 4.63 | 0.23 | -2.86986 | 0.045593 | 0.992011 | Down |
| NC_019476.2:42985455\|42994446 | 101108429 | 3.4 | 0 | -3.19322 | 0.066964 | 0.992011 | Down |
| NC_019482.2:43799741\|43805516 | 101120136 | 4.43 | 0.35 | -2.47314 | 0.086166 | 0.992011 | Down |
| NC_019478.2:42880385\|42880571 | n/a | 3.06 | 0 | -3.076 | 0.085529 | 0.992011 | Down |
| NC_019459.2:248638951\|248652113 | 101110912 | 11.24 | 2.55 | -1.23463 | 0.08959 | 0.992011 | Down |
| NC_019458.2:176469280\|176474355 | 101115505 | 3.65 | 0.21 | -2.64809 | 0.068202 | 0.992011 | Down |
| NC_019460.2:5041303\|5051621 | 101121164 | 7.64 | 1.94 | -1.24959 | 0.091647 | 0.992011 | Down |
| NC_019464.2:99559837\|99566690 | 101115969 | 7 | 1.5 | -1.54014 | 0.098021 | 0.992011 | Down |
| NC_019461.2:111287336\|111287760 | 101106584 | 3.12 | 0 | -3.01679 | 0.050872 | 0.992011 | Down |
| NC_019461.2:104568896\|104582100 | 101108220 | 5.34 | 0.91 | -2.48787 | 0.053374 | 0.992011 | Down |
| NC_019464.2:79447120\|79457089 | 101103403 | 7.07 | 8.39 | 1.135802 | 0.073882 | 0.992011 | Up |
| NC_019460.2:205411615\|205482394 | n/a | 1.84 | 3.24 | 1.478382 | 0.089158 | 0.992011 | Up |
| NC_019459.2:117036184\|117046567 | 101109858 | 4.67 | 0.8 | -2.52616 | 0.025967 | 0.992011 | Down |
| NC_019466.2:59497675\|59499868 | 101120338 | 0.47 | 1.52 | 2.654687 | 0.075481 | 0.992011 | Up |
| NC_019462.2:43091865\|43096948 | 101101980 | 0.47 | 2.7 | 3.424418 | 0.002253 | 0.992011 | Up |
| NC_019458.2:215109570\|215118738 | 101118840 | 4.44 | 5.17 | 1.300899 | 0.064151 | 0.992011 | Up |
| NC_019460.2:44203819\|44204892 | 101115777 | 2.59 | 0 | -3.07248 | 0.086373 | 0.992011 | Down |
| NC_019464.2:83840861\|83845941 | 101110944 | 4.09 | 4.96 | 1.440586 | 0.086694 | 0.992011 | Up |
| NC_019466.2:22083032\|22087150 | 101121432 | 2.76 | 0.2 | -2.57704 | 0.082361 | 0.992011 | Down |
| NC_019470.2:78719744\|78766679 | 101119239 | 0.28 | 2.38 | 3.261351 | 0.034201 | 0.992011 | Up |
| NC_019460.2:195031175\|195052666 | 101121336 | 5.61 | 0.55 | -2.81938 | 0.030503 | 0.992011 | Down |
| NC_019464.2:33216633\|33224910 | 101114616 | 2.69 | 0 | -3.18862 | 0.073156 | 0.992011 | Down |
| NC_019458.2:267172147\|267194429 | 101116444 | 6.79 | 1.94 | -1.42727 | 0.06992 | 0.992011 | Down |
| NC_019458.2:76508042\|76510961 | 101112360 | 11.99 | 3.34 | -1.21159 | 0.075113 | 0.992011 | Down |
| NC_019470.2:59737796\|59761429 | 101121957 | 7.1 | 1.07 | -1.7306 | 0.068404 | 0.992011 | Down |
| NC_019482.2:856579\|871272 | 101102120 | 0.33 | 1.72 | 2.680491 | 0.094195 | 0.992011 | Up |
| NC_019459.2:235665695\|235670535 | 101121575 | 0.42 | 1.74 | 2.936158 | 0.052104 | 0.992011 | Up |
| NC_019473.2:38047295\|38051993 | 101109911 | 5.25 | 0.45 | -2.35012 | 0.041333 | 0.992011 | Down |
| NC_019484.2:13099077\|13099679 | 101102792 | 16.26 | 4.85 | -1.16936 | 0.094583 | 0.992011 | Down |
| NC_019477.2:38628056\|38635982 | 101123671 | 0.75 | 2.48 | 2.75179 | 0.056628 | 0.992011 | Up |
| NC_019458.2:233343283\|233354436 | 101114918 | 4.7 | 0.57 | -2.13908 | 0.056841 | 0.992011 | Down |
| NC_019467.2:81605219\|81607819 | 101115718 | 5.55 | 5.15 | 1.13215 | 0.089051 | 0.992011 | Up |
| NC_019466.2:81907600\|81908115 | 101109367 | 0.83 | 1.67 | 2.394693 | 0.093909 | 0.992011 | Up |
| NC_019459.2:51983787\|51992132 | 101110301 | 1.05 | 2.49 | 2.056113 | 0.084983 | 0.992011 | Up |
| NC_019458.2:164084583\|164088877 | 101111506 | 7.97 | 0.86 | -2.30378 | 0.019986 | 0.992011 | Down |
| NC_019459.2:42843788\|42844339 | 101114752 | 0.9 | 2.67 | 2.761478 | 0.02408 | 0.992011 | Up |
| NC_019458.2:201030000\|201031065 | 101122320 | 2.67 | 0 | -2.90445 | 0.088001 | 0.992011 | Down |
| NC_019471.2:43812560\|43813830 | 101111736 | 0.4 | 2.39 | 3.49798 | 0.004334 | 0.992011 | Up |
| NC_019471.2:50356474\|50392487 | n/a | 3.68 | 5.12 | 1.690137 | 0.068162 | 0.992011 | Up |
| NC_019463.2:58866770\|58868633 | 101114956 | 2.97 | 0 | -2.92432 | 0.083996 | 0.992011 | Down |
| NC_019464.2:99559837\|99596346 | 101115969 | 3.33 | 0.24 | -2.24965 | 0.060822 | 0.992011 | Down |
| NC_019479.2:17080988\|17109247 | 101112014 | 0 | 1.22 | 3.202028 | 0.089771 | 0.992011 | Up |
| NC_019459.2:33659958\|33673647 | 101115255 | 5.68 | 0.78 | -2.18325 | 0.097651 | 0.992011 | Down |
| NC_019459.2:244595100\|244603200 | 101105220 | 0.31 | 1.33 | 2.992574 | 0.063805 | 0.992011 | Up |
| NC_019461.2:32288900\|32299052 | 101111186 | 4.59 | 0 | -3.51056 | 0.020717 | 0.992011 | Down |
| NC_019464.2:48212845\|48216051 | 101115967 | 2.94 | 5.69 | 1.644205 | 0.043106 | 0.992011 | Up |
| NC_019458.2:245223671\|245227738 | 101108362 | 5.19 | 0.78 | -2.0612 | 0.053515 | 0.992011 | Down |
| NC_019463.2:89517637\|89518332 | 101104239 | 2.28 | 3.86 | 1.634981 | 0.073717 | 0.992011 | Up |
| NC_019461.2:32735867\|32814436 | 101122517 | 4.37 | 0.58 | -2.13639 | 0.075674 | 0.992011 | Down |
| NC_019463.2:37361524\|37375034 | 101104320 | 5.27 | 1.32 | -1.95934 | 0.070037 | 0.992011 | Down |
| NC_019459.2:137149076\|137156566 | 101116207 | 4.59 | 0.75 | -1.75703 | 0.096187 | 0.992011 | Down |
| NW_014641849.1:18611\|22853 | 105605978 | 8.43 | 0 | -4.33483 | 0.001806 | 0.992011 | Down |
| NC_019464.2:51974463\|51979853 | 101122791 | 7.55 | 0.99 | -2.39156 | 0.033509 | 0.992011 | Down |
| NW_014639656.1:7357\|13617 | n/a | 0 | 1.18 | 3.42066 | 0.083395 | 0.992011 | Up |
| NC_019463.2:66658810\|66666380 | 101102237 | 3.53 | 0 | -3.54522 | 0.01189 | 0.992011 | Down |
| NC_019470.2:62736099\|62741891 | 101111476 | 0.41 | 1.77 | 2.681722 | 0.07061 | 0.992011 | Up |
| NC_019459.2:160176011\|160228482 | 106990963 | 11.35 | 2.7 | -1.44756 | 0.080267 | 0.992011 | Down |

**Supplementary Figure 1:** Representative sequencing quality control profiles of circRNA libraries from Tibetan sheep ovaries.


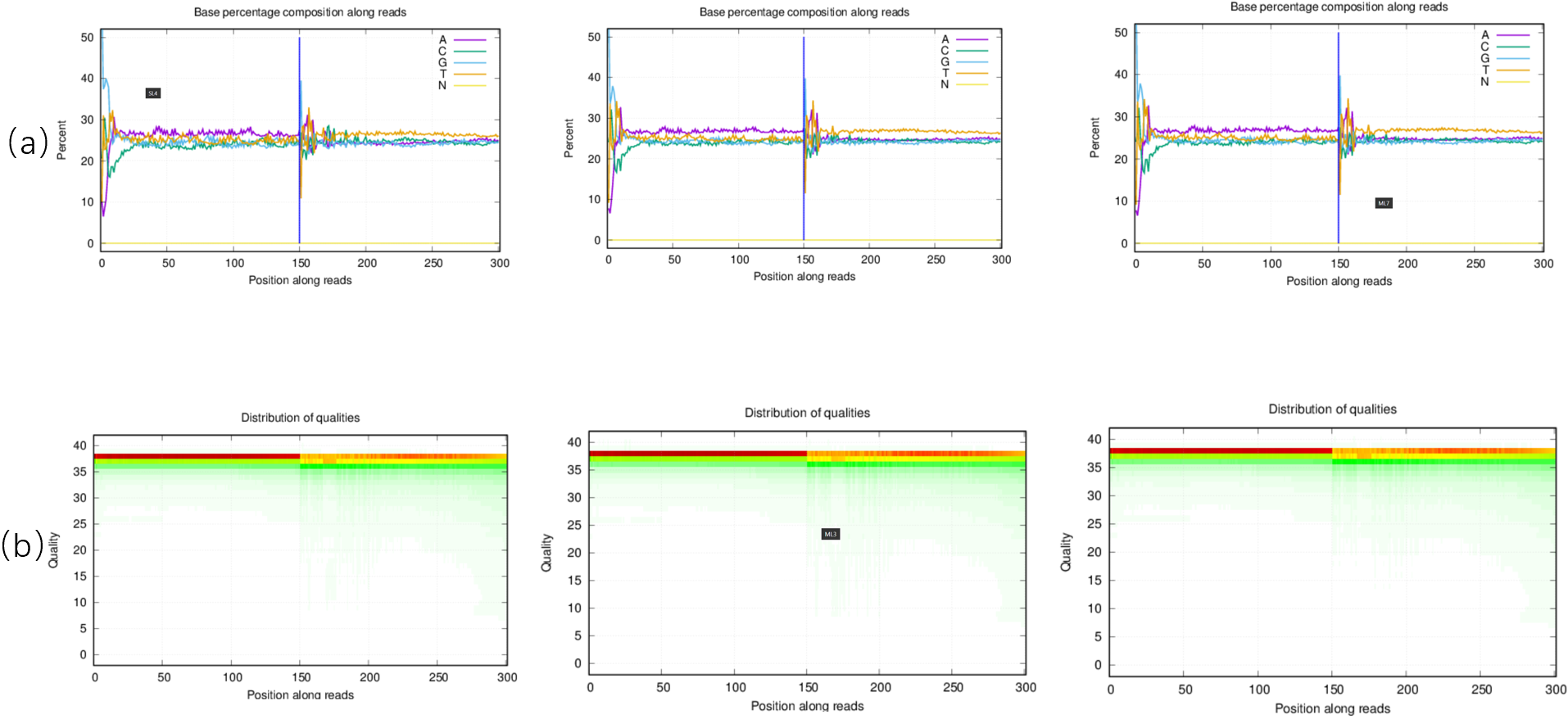


(A) Per-base sequence quality scores (Fastp). Phred scores > 30 across all bases.

(B) Base percentage composition along reads (Fastp), showing expected random primer bias in the first ~12 bp and at the R1/R2 junction, with stable distribution thereafter. Similar patterns were observed in all 20 libraries.

**Supplementary Table 3:** PPI network key node degree value.

| Network | Hub Gene | Degree Value |
| --- | --- | --- |
| Upregulated | EP300 | 16 |
| Upregulated | TCF7 | 12 |
| Upregulated | RNF111 | 10 |
| Upregulated | E2F2 | 9 |
| Upregulated | LSM14A | 8 |
| Downregulated | CUL1 | 18 |
| Downregulated | CSNK2A1 | 14 |
| Downregulated | CDC42 | 11 |
| Downregulated | RAP1B | 10 |
| Downregulated | MAP3K2 | 9 |
